# Fifteen compelling open questions in plant cell biology

**DOI:** 10.1093/plcell/koab225

**Published:** 2021-09-16

**Authors:** Adrienne H K Roeder, Marisa S Otegui, Ram Dixit, Charles T Anderson, Christine Faulkner, Yan Zhang, Maria J Harrison, Charlotte Kirchhelle, Gohta Goshima, Jeremy E Coate, Jeff J Doyle, Olivier Hamant, Keiko Sugimoto, Liam Dolan, Heather Meyer, David W Ehrhardt, Arezki Boudaoud, Carlos Messina

**Affiliations:** Weill Institute for Cell and Molecular Biology and School of Integrative Plant Science, Section of Plant Biology, Cornell University, Ithaca, New York 14853, USA; Department of Botany and Center for Quantitative Cell Imaging, University of Wisconsin-Madison, Wisconsin 53706, USA; Department of Biology and Center for Engineering Mechanobiology, Washington University in St Louis, Missouri 63130, USA; Department of Biology and Center for Lignocellulose Structure and Formation, The Pennsylvania State University, University Park, Pennsylvania 16802, USA; Crop Genetics, John Innes Centre, Norwich Research Park, Norwich NR4 7UH, UK; State Key Laboratory of Crop Biology, College of Life Sciences, Shandong Agricultural University, Tai’an, China; Boyce Thompson Institute, Ithaca, New York 14853, USA; Department of Plant Sciences, University of Oxford, Oxford OX1 3RB, UK; Laboratoire de Reproduction et Développement des Plantes, Université de Lyon, ENS de Lyon, UCBL, INRAE, CNR S, Lyon Cedex 07, France; Sugashima Marine Biological Laboratory, Graduate School of Science, Nagoya University, Nagoya, Japan; Department of Biology, Reed College, Portland, Oregon 97202, USA; School of Integrative Plant Science, Section of Plant Biology and Section of Plant Breeding and Genetics, Cornell University, Ithaca, New York 14853, USA; Laboratoire de Reproduction et Développement des Plantes, Université de Lyon, ENS de Lyon, UCBL, INRAE, CNR S, Lyon Cedex 07, France; Center for Sustainable Resource Science, RIKEN, Kanagawa 230-0045, Japan; Department of Biological Sciences, Graduate School of Science, The University of Toky o, Tokyo 113-0033, Japan; Gregor Mendel Institute of Molecular Plant Biology GmbH, Vienna 1030, Austria; Department of Plant Biology, Carnegie Institution for Science, Stanford, California 94305, USA; Department of Plant Biology, Carnegie Institution for Science, Stanford, California 94305, USA; LadHyX, CNRS, Ecole Polytechnique, Institut Polytechnique de Pari s, Palaiseau Cedex 91128 France; Corteva Agriscience, Johnston, Iowa 50310, USA

## Abstract

As scientists, we are at least as excited about the open questions—the things we do not know—as the discoveries. Here, we asked 15 experts to describe the most compelling open questions in plant cell biology. These are their questions: How are organelle identity, domains, and boundaries maintained under the continuous flux of vesicle trafficking and membrane remodeling? Is the plant cortical microtubule cytoskeleton a mechanosensory apparatus? How are the cellular pathways of cell wall synthesis, assembly, modification, and integrity sensing linked in plants? Why do plasmodesmata open and close? Is there retrograde signaling from vacuoles to the nucleus? How do root cells accommodate fungal endosymbionts? What is the role of cell edges in plant morphogenesis? How is the cell division site determined? What are the emergent effects of polyploidy on the biology of the cell, and how are any such “rules” conditioned by cell type? Can mechanical forces trigger new cell fates in plants? How does a single differentiated somatic cell reprogram and gain pluripotency? How does polarity develop de-novo in isolated plant cells? What is the spectrum of cellular functions for membraneless organelles and intrinsically disordered proteins? How do plants deal with internal noise? How does order emerge in cells and propagate to organs and organisms from complex dynamical processes? We hope you find the discussions of these questions thought provoking and inspiring.

## Teaser

We asked 15 experts to address what they consider to be the most compelling open questions in plant cell biology and these are their questions.

## Introduction

### (Written by Adrienne H. K. Roeder, editor)

Science happens in the interface between known and unknown and asking hard questions plays a key part in the scientific process (Siegfried, 2005). In this article, we focus on the unknown, the mysteries waiting to be solved. We asked 15 experts to each write a short description of the biggest open question in their subfield of plant cell biology and compiled their answers here. Some questions are age-old and have been studied for decades with great progress, yet each result opens deeper mysteries. Some questions are newer, arising from a recent recognition of importance. We hope this collection of questions piques your curiosity, stimulates your interest in new topics, and maybe even generates ideas for future research. No list is ever complete. With only 15 questions, we have missed many other important mysteries. Please let us know your favorite open plant cell biology questions in the Plantae discussion (https://plantae.org/what-are-the-big-open-questions-in-plant-cell-biology) for this article.

## How are organelle identity, domains, and boundaries maintained under the continuous flux of vesicle trafficking and membrane remodeling?

### (Written by Marisa S. Otegui)

**Figure 1:**
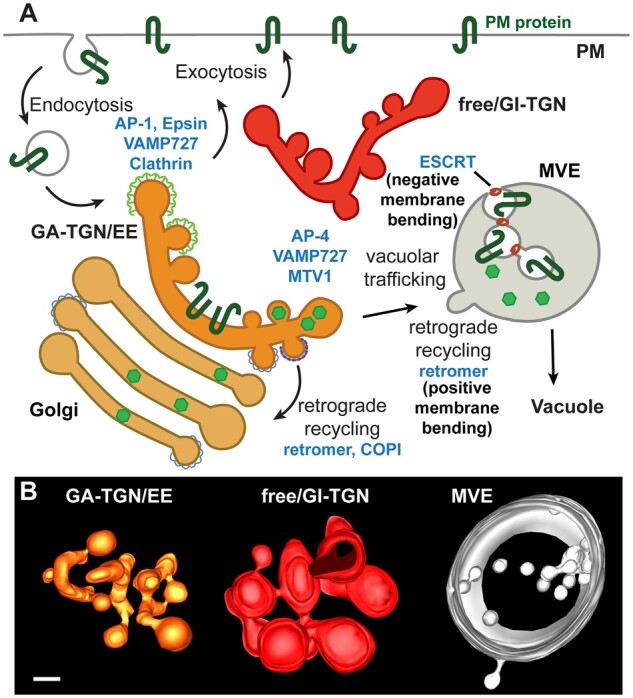
Vesicular trafficking and plant endosomes. A, Diagram showing the main vesicular trafficking pathways involving the TGN and MVEs. The main coats components and factors involved in vesicle formation are in blue. The two “zones” of the Golgi-associated TGN/early endosome (GA-TGN/EE) are characterized by either AP-1, Epsin, VAMP727, and clathrin (exocytosis/secretion trafficking zone) or APP-4, VAMP727, MTV1 (vacuolar trafficking zone). Small green hexagons depict soluble vacuolar cargo transported through the vacuolar trafficking zone of the TGN to MVEs and to the vacuole. At MVEs, intralumenal vesicles containing endocytosed PM proteins targeted for vacuolar degradation are formed by the action of ESCRT proteins and recycling vesicles bud into the cytoplasm, likely coated with the retromer complex. B, Electron tomographic reconstructions of a Golgi-associated TGN/early endosome (GA-TGN/EE), a free/Golgi-independent TGN (free/GI-TGN), and an MVE from a root plant cell. Scale bar = 50 nm.

One of the most fascinating topics in plant cell biology is the regulation of vesicular trafficking through the endomembrane system. In the secretory pathway, newly synthesized cargo proteins typically are carried in vesicles from the endoplasmic reticulum (ER), to the Golgi, to the trans-Golgi Network (TGN), and from there, either to the plasma membrane (PM; exocytosis/secretion) or to the vacuole. In the endocytic pathway, PM proteins are sorted into endocytic vesicles and delivered first to the TGN (also called the early endosome for its function in the endocytic pathway), where they can be recycled to the PM or further carried to multivesicular endosomes (MVEs) for further sorting into intralumenal vesicles and degradation in the vacuolar lumen (Figure 1; Paez-Valencia et al., 2016). The retrograde pathways also generate vesicles to recycle components to their original donor compartment. Vesicle trafficking is essential to plant development and environmental responses; mutations in genes coding for the core factors regulating membrane trafficking often lead to severe developmental abnormalities or even lethality (Paez-Valencia et al., 2016).

Vesicle formation requires the concentration of cargo proteins in a small domain of the donor membrane and cytosolic proteins/coats with the ability to bend that membrane domain into a vesicle. Three conserved protein coat systems, Coat Protein Complex I (COPI), COPII, clathrin (Bonifacino and Lippincott-Schwartz, 2003), as well as the retromer complex (Burd and Cullen, 2014), mediate the formation of vesicles and tubules through positive membrane bending (budding into the cytoplasm), whereas endosomal sorting complex required for transport (ESCRT) proteins facilitate membrane budding in the opposite topology, away from the cytoplasm (Vietri et al., 2020). The COPI coat mediates intra-Golgi and retrograde Golgi-ER trafficking, COPII-coated vesicles form at the ER exit sites and mediate anterograde transport to the Golgi, and clathrin is recruited mostly to the PM and TGN by specific adaptor complexes and other accessory factors (Bonifacino and Lippincott-Schwartz, 2003). The retromer functions in retrograde recycling from endosomes, whereas ESCRT proteins mediate intralumenal vesicle formation also in endosomes. Thus, the same organelles can produce different types of vesicles, from both the retrograde and anterograde pathways, and even in opposite topologies. How are these budding domains physically segregated but at the same time tightly integrated to maintain the stable identity of the endomembrane system? This is a largely unanswered and puzzling question. Some of the most striking examples of organelles with complex and spatially segregated budding activities are found in the TGN and MVEs.

The TGN and MVEs have a short life, from minutes to few hours depending on the cell type. The TGN derives from the *trans*-most cisterna, detaches from the Golgi as an independent organelle and finally is fragmented into multiple vesicles (Toyooka et al., 2009; Kang et al., 2011; Uemura et al., 2014, 2019), whereas MVEs partially derive from TGN-associated membranes and fuse with the vacuole to release their internal vesicles into the vacuolar lumen. Thus, plant cells contain subpopulations of TGNs at different maturation stages and with distinct trafficking capabilities (Renna et al., 2018; Uemura et al., 2019; Ito and Boutté, 2020). As part of its endosomal functions, the TGN receives and recycles PM cargo whereas as part of the secretory pathway, it mediates retrograde recycling through COPI- (Bykov et al., 2017) and retromer-mediated (Niemes et al., 2010) vesiculation, secretory vesicles destined to the PM and vesicles carrying vacuolar cargo (Rosquete et al., 2018; Figure 1).

How all these functions are coordinated and segregated within the TGN is largely unknown. Some functions may be specific to different TGN populations. For example, only Golgi-associated but not free Golgi-independent TGNs are stained by the endocytic tracer FM4–64, whereas free TGNs are mostly involved in exocytosis (Uemura et al., 2019). Recent studies have shown that subdomains with different protein and lipid compositions co-exist within the same TGNs (Wattelet-Boyer et al., 2016). For example, Golgi-associated TGNs have at least two “zones”: the secretory-trafficking zone that forms vesicles destined to the PM and is enriched in the soluble *N*-ethylmaleimide-sensitive factor attachment protein receptor (SNARE) VAMP721, the adaptor complex AP-1, the accessory protein EPSIN1, and clathrin, and the vacuolar trafficking zone enriched in the SNARE VAMP727, the adaptor complex AP-4, and accessory protein MODIFIED TRANSPORT TO THE VACUOLE1 (Heinze et al., 2020; Shimizu et al., 2021). Whether the secretory-trafficking zone also mediates the recycling of endocytosed proteins back to the PM is currently unknown but at least it is clear that there is more than one pathway controlling exocytosis from the TGN, as the TGN-localized protein ECHIDNA controls the secretion of only a subset of PM proteins (Boutté et al., 2013) whereas a complex consisting of seven transmembrane domain-containing proteins and guanine nucleotide-binding protein signaling components regulate exocytosis of cellulose synthases but general exocytosis of other cargo (McFarlane et al., 2021).

At MVEs, budding occur in two opposite directions, toward the cytoplasm to form retromer-mediated recycling vesicles and tubules, and into the lumen to generate ESCRT-mediated intralumenal vesicles containing PM proteins sorted for vacuolar degradation (Norris and Grant, 2020; Figure 1). In animal cells, the segregation and coordination between these two opposite budding activities seem to include interactions between the retromer and ESCRT machineries that cross-regulate their assembly and disassembly (Norris and Grant, 2020). However, these mechanisms remain poorly understood. It is also unclear whether plant MVEs could be regulated in similar ways, as plant MVE recycling activity has not been fully characterized, and ESCRT-mediated inward budding in plant endosomes seem to differ from other organisms. In plants, membrane constriction at the neck of the forming bud is uncoupled from membrane scission, leading to the formation of concatenated intralumenal vesicles connected by membranous bridges (Buono et al., 2017; Goodman et al., 2021; Figure 1).

Whereas it is clear that cargo concentration and recruitment of specific coat components and membrane bending proteins are critical for vesicle formation, how adjacent budding subdomains within a single organelle are established and coordinated remains poorly understood. Membrane curvature can be driven through structured membrane-bending protein scaffolds, such as amphipathic helices that insert into one of the membrane leaflets or through inherently curved protein domains. In addition, in recent years, it has been demonstrated that membrane curvature can be also driven by protein phase separation and formation of liquid-like assemblies by cytosolic proteins with intrinsically disordered domains (Yuan et al., 2021). Ongoing and future research will determine whether protein-based phase separation could also play a role in the segregation of budding domains within organelles.

Although questions related to organelle identity and membrane domain segregation have intrigued cell biologists for a long time, new technologies such as proteomic profiling of single organelles or even sub-domains within organelles, imaging by super-resolution and/or cryo-electron microscopy, and the possibility to generate mutant of single or multiple uncharacterized genes by CRISPR-based approaches are bringing the possibility to understand the underlying molecular intricacies of membrane trafficking in plant cells.

### Funding

Work on endosomal trafficking in the Otegui Lab is supported by grant National Science Foundation (NSF) MCB2114603 to MSO.

## Is the plant cortical microtubule cytoskeleton a mechanosensory apparatus?

### (Written by Ram Dixit)

Plants experience a variety of intrinsic and extrinsic mechanical signals that can differ in magnitude, direction, and duration. Physical forces propagate over considerable distances in solid tissues by contrast to diffusible chemical signals which dissipate rather quickly. The speed of transmission of mechanical information is also faster than chemically encoded information (Na et al., 2008). Therefore, mechanical signals are ideal for long-range and rapid coordination of cell behavior during plant growth and development. Diverse sensory mechanisms operate at the molecular, cellular, and organ levels to help plants interpret their mechanical environment (Hamant and Haswell, 2017).

While the cortical microtubule cytoskeleton is best known for defining the axis of cell expansion by orienting cell wall deposition, there is growing appreciation for its responsiveness to mechanical stimuli. Imposing or altering mechanical stress causes cortical microtubules to align along the direction of maximal stress. In both plants and animals, the cytoskeleton is typically considered as a downstream target of signaling. Here, I emphasize a force-sensing function for the cortical microtubule cytoskeleton that can detect and integrate mechanical signals. A mechanosensory role for cortical microtubules might explain why mature cells that have ceased expanding invest considerable energy in creating and maintaining cortical microtubules.

Plant cells are cemented together by their cell walls. In each cell, the cell wall is attached to the PM, and in turn the PM attaches to cortical microtubules along their length. In addition, there might exist transmembrane proteins that directly link cortical microtubules to cell wall components. Together, these physical connections can mechanically couple the cell wall, PM, and cortical microtubules. This could allow cortical microtubules to directly sense mechanical stress from the cell wall (Hamant et al., 2019), which would enable cells to quickly perceive dynamically changing mechanical inputs.

**Figure 2:**
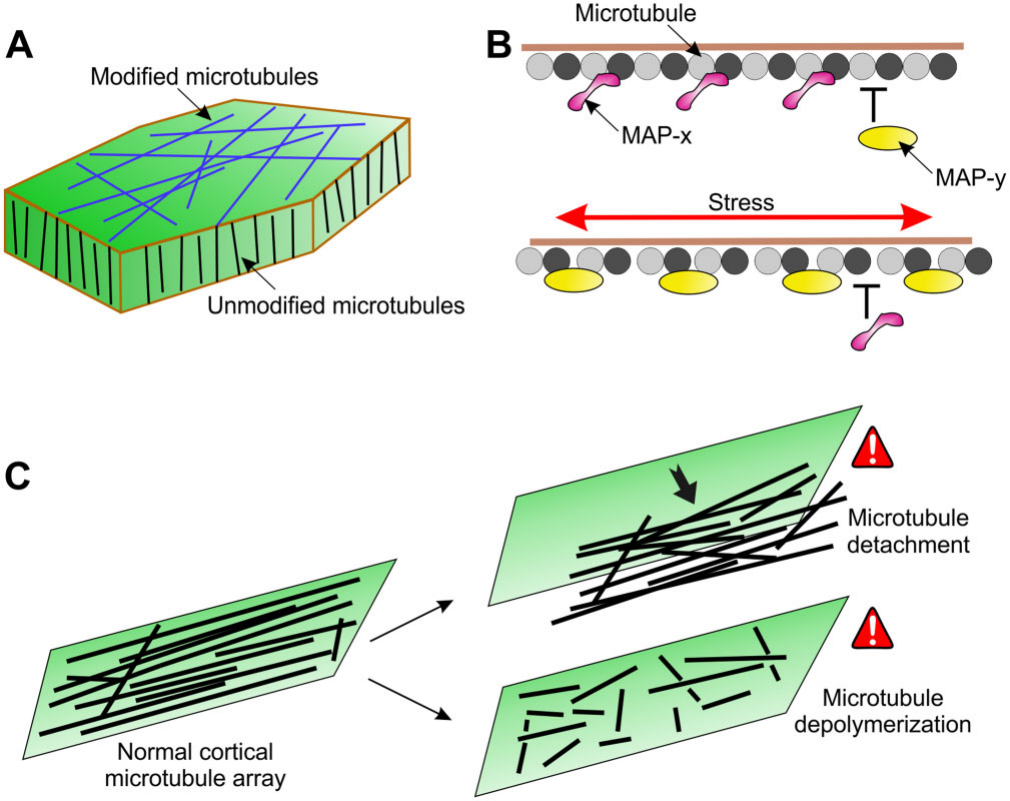
Cortical microtubules as mechanosensors. A, Regulation of the alignment and density of cortical microtubules can impact their ability to sense mechanical signals. Modification of cortical microtubules (blue) by microtubule-associated proteins and tubulin posttranslational modifications provides a potential mechanism to locally control force-sensitivity of cortical microtubules. B, Disruption of the microtubule lattice by mechanical stress (red arrow) might affect protein interactions to directly transduce force into a biochemical signal. In this example, a hypothetical protein MAP-x preferentially binds to unstretched microtubules, whereas a hypothetical protein MAP-y preferentially binds to stretched microtubules. For simplicity, microtubules are shown as a single protofilament of tubulin dimers. C, Sensors are proposed to perceive severe cortical microtubule perturbations such as detachment from the PM and extensive depolymerization or fragmentation to initiate cortical microtubule integrity signaling.

If the cell wall, PM, and cortical microtubules are mechanically coupled, then modulating their material properties and linkages provides the means to tune the transmission and interpretation of mechanical signals. Changes in turgor pressure due to developmental and environmental signals will alter the turgor-induced mechanical stress within the cell wall. In addition, cells actively modify the cell wall by stiffening, loosening, and/or breaking fibrils to regulate the cell wall mechanical stress. Similarly, cells might actively modify the physical properties of cortical microtubules to tune their force-sensing ability. Regulating the attachment of cortical microtubules to the PM, their alignment, and density would affect the way microtubules sense mechanical signals. In addition, microtubule-associated proteins and tubulin posttranslational modifications might provide nuanced control of the mechanical properties of cortical microtubules to fine-tune their force sensitivity. For example, MAP65 (Microtubule-Associated Protein of 65 kDa) proteins increase microtubule flexibility (Portran et al., 2013), whereas the mammalian MAP2 (Microtubule-Associated Protein 2) and tau proteins increase microtubule rigidity (Felgner et al., 1997). By spatially varying the microstructure of the cortical microtubule cytoskeleton, a cell could create a functionally graded sensory structure (Figure 2A).

How might microtubules transduce mechanical signals? One possibility is that mechanical stress deforms the microtubule lattice, exposing normally inaccessible tubulin sites (Figure 2B). This might cause other proteins to bind or unbind microtubules to transduce the signal. If the extent of microtubule lattice deformation is a function of the magnitude and direction of force, this would provide a mechanism to decode the quality and quantity of mechanical signals. Since externally applied force often elicits a Ca^2+^ and/or pH response, it will be interesting to determine whether the magnitude of applied force correlates positively with the amount and/or duration of a Ca^2+^ or pH response in a microtubule-dependent manner. Another, not mutually exclusive, possibility is that microtubule deformation triggers signaling by regulating the activity of ion channels, transmembrane receptors, and/or gene expression through Linker of Nucleoskeleton and Cytoskeleton complexes.

In vitro optical trapping experiments can be used to determine whether stretching of microtubules directly affects protein interactions. For example, beads attached to a microtubule in vitro can be used to exert tensile force on the microtubule using a dual-beam laser trap and the binding/unbinding of specific proteins monitored under these conditions. Such experiments would establish the relationship between magnitude of applied force and extent of binding of specific microtubule-associated proteins in vitro. Subsequently, the level of binding of these proteins to cortical microtubules when cells are subjected to mechanical force could be used to estimate the magnitude of stress on cortical microtubules in vivo and to examine whether turgor pressure affects the force-sensing ability of microtubules. Based on available data, MAP65 and katanin are promising candidates as tension-sensitive microtubule-associated proteins. In addition, it would be interesting to study the effect of microtubule stretching on the binding of signaling components such as Rho of plants (ROP)-interactive CRIB motif-containing protein 1 and Never in Mitosis A (NIMA)-related kinase 6. Characterization of a force-sensitive microtubule-associated protein would open the door to identifying other such proteins, as demonstrated recently for the force-induced actin-binding protein, zyxin, using proximity biotinylation (Cheah et al., 2021). Another exciting outcome of this type of work would be the identification of force-sensing domains that could be used to develop biosensors to visualize the spatial distribution of forces in living cells.

Adaptation is an important characteristic of sensory systems to be able to sense new information. Interestingly, recent work suggests that cortical microtubules require changes in tension to respond (Colin et al., 2020), suggesting that they acclimate to mechanical stimuli. The dynamic nature of cortical microtubules primes the system to sense changes in their mechanical environment. The rotary movement of cortical microtubules (Chan et al., 2007) is particularly intriguing as a potential mechanism to vary cortical microtubule organization to sense new mechanical cues. One possibility is that acclimation to mechanical stress feeds back on cortical microtubule rotary movement, which predicts that the microtubule rotary behavior will be significantly different between unloaded control cells and cells experiencing either uniform or fluctuating mechanical stress.

The mechanosensory (and cell wall deposition) function of the cortical microtubule cytoskeleton depends on its structural integrity. Analogous to cell wall integrity signaling, it is plausible that cortical microtubule integrity signaling exists for the maintenance of the cortical microtubule cytoskeleton upon damage by adverse environmental and physiological conditions (Figure 2C). For example, drought and salt stress rapidly induce depolymerization and disorganization of cortical microtubules. However, this is transient, and cells subsequently restore the cortical microtubule polymer mass and organization. While some potential signaling components have been identified (Fujita et al., 2013; Bhaskara et al., 2017), the sensors and effectors that would constitute cortical microtubule integrity signaling remain elusive. Mature cells, in which the cell wall deposition function of cortical microtubules might not be dominant, could be good model systems to examine this process.

### Acknowledgments

My apology to colleagues whose work could not be cited due to length restrictions.

### Funding

This work was supported by the Center for Engineering Mechanobiology, a National Science Foundation Science and Technology Center, under grant agreement CMMI: 15-48571 and National Institute of General Medical Sciences of the National Institutes of Health under award number R35GM139552.

## How are the cellular pathways of cell wall synthesis, assembly, modification, and integrity sensing linked in plants?

### (Written by Charles T. Anderson)

**Figure 3:**
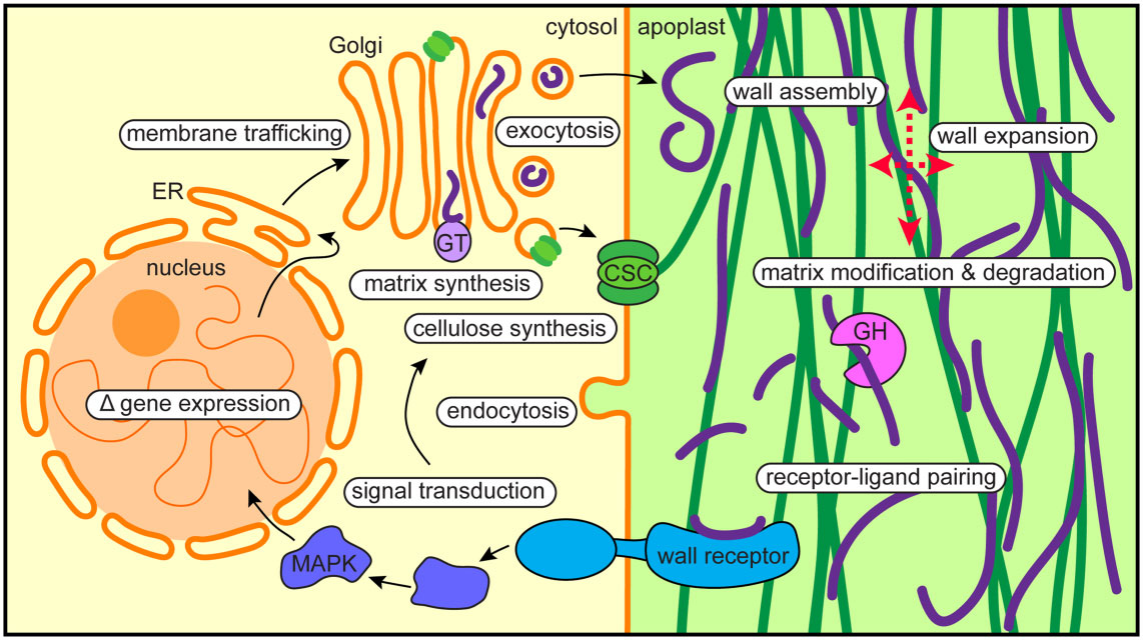
Potential links between intracellular and extracellular cell wall regulation in growing plant cells. Transcription of cell wall-related genes in the nucleus, followed by mRNA export and translation on the rough ER surface, leads to the production of soluble and membrane proteins that enter the anterograde membrane trafficking system. In the Golgi, matrix polysaccharides including pectins and hemicelluloses are synthesized by large suites of glycosyltransferases (GTs) and other enzymes. Matrix polysaccharides and cellulose synthesis complexes (CSCs) are trafficked from the Golgi to the cell surface, where matrix polysaccharides (purple) are exocytosed into the apoplast and CSCs extrude cellulose (green) into the wall. These polymers and glycoproteins assemble into a strong, flexible wall that can expand anisotropically (more in one direction than another, see red dashed arrows), and matrix polysaccharides are modified in the wall and can be degraded by glycosyl hydrolases/lyases (GH). Wall polymer fragments can bind to wall receptors, which initiate intracellular signaling cascades that can change gene expression or modulate wall synthesis and/or assembly through trafficking and posttranslational modification of relevant proteins. Compartments/proteins are labeled with normal text and processes are highlighted in white ovals; arrows can entail multiple events/processes. MAPK, mitogen-activated protein kinase.

Plant cells construct walls around themselves that protect and support them, adhere them to neighboring cells, and mold cell and organ morphogenesis to generate an amazing diversity of forms. The walls of growing plant cells are complex, dynamic structures that are composed of interacting networks of polysaccharides and glycoproteins, as well as enzymes, metabolites, and water (Anderson and Kieber, 2020). Many wall polymers are synthesized intracellularly, trafficked to the cell surface, and secreted into the apoplast (Figure 3), although cellulose, the toughest component of growing walls, is extruded directly into the apoplast. A large number of wall-relevant genes in plant genomes, combined with the bewildering complexity of polymer synthesis, intracellular transport, secretory, and apoplastic events that are required to build and maintain a wall that is both strong and flexible, means that we have barely scratched the surface of understanding how the cell wall is assembled, perceived, and monitored by the protoplast it contains, and remodeled during growth. How these processes are regulated and interlinked by cell-surface or intracellular receptors, signal transduction, changes in gene expression, and protein translation, trafficking, and posttranslational modification are an even bigger mystery (Figure 3).

Progress in structural biology, biochemistry, and cell biology has started to reveal the molecular and cellular mechanisms by which plant cells assemble their walls (Anderson and Kieber, 2020), and technical advances including mass spectrometry (Voxeur et al., 2019) and solid-state Nuclear Magnetic Resonance spectroscopy (NMR) (Zhao et al., 2020) are beginning to decipher the detailed structures and interactions of wall polysaccharides, which can be almost infinitely complex due to the fact that these polymers are biochemically synthesized rather than genetically templated. This complexity suggests that wall polymers can contain unique molecular signatures that drive specific and tunable polysaccharide–polysaccharide and polysaccharide–protein (Francoz et al., 2019) interactions, so that much like an individual person, each wall molecule can be thought of as a unique entity, be tracked over time and space, and be contextualized based on its surroundings and its interactions with those surroundings.

The cell wall provides an ever-changing flow of physical and molecular information to its encapsulated cell. Our current understanding of the processes by which plant cells capture and interpret this information, dubbed wall integrity sensing (Vaahtera et al., 2019), has benefitted from studies of plant–pathogen interactions, in which pathogens degrade wall components as they invade the plant (Molina et al., 2021) to produce damage-associated molecular patterns. Additionally, plants degrade their own walls during growth, and some of these fragments are thought to bind to receptors that trigger intracellular signaling to maintain wall homeostasis (Feng et al., 2018), but the exact identity of wall-derived ligands and the extent to which autogenously generated wall fragments (Yang et al., 2021) provide recycled materials for new wall synthesis (Barnes and Anderson, 2018) and influence intracellular signaling, gene expression, and wall biosynthetic and assembly pathways is poorly understood. Much remains to be learned regarding how the cell wall “code” is translated into information that the cell perceives, responds to, and edits.

In considering the life history of each plant cell wall, we can ask to what extent its structure and composition have changed during cell growth and to what extent it has become “fossilized,” with older wall layers potentially influencing the deposition patterns of new layers (Chan and Coen, 2020). Exploring this stratified information at the nanoscale after experimentally manipulating the plant’s environment, metabolism, and/or cell biological processes might yield clues into how plant cells respond to stimuli both intracellularly and extracellularly to reshape their walls and optimize survival, growth, and reproduction, but these analyses will be challenging due to the high density and diversity of wall polymers. Viewing individual cell walls as analogous to rock cores or tree rings and applying the analytical and conceptual tools of geological stratigraphy and dendrochronology/climatology, metabolic labeling techniques that allow for tracing sub-populations of wall polymers and new imaging probes for plant cell walls (DeVree et al., 2021), might be used to provide new insights into cell wall dynamics.

These are exciting times for the cell biological study of plant cell walls. Identifying and closing the loops between intracellular biosynthesis, wall assembly, wall modification and degradation, receptor-mediated perception of wall degradation products, signal transduction, and gene/mRNA/protein-level regulatory networks will provide a clearer picture of how plants construct dynamic and strong cell walls that both underpin their development and provide us with useful biomass that has been distilled from the air using sunlight and water.

### Acknowledgments

Thanks to members of the Anderson Lab and the Center for Lignocellulose Structure and Formation for inspiring discussions, and apologies to those whose important contributions could not be cited.

### Funding

This work was supported as part of The Center for Lignocellulose Structure and Formation, an Energy Frontier Research Center funded by the US Department of Energy, Office of Science, Basic Energy Sciences under Award # DE-SC0001090.

## Why do plasmodesmata open and close?

### (Written by Christine Faulkner)

Like all multicellular organisms, plants rely on short and long-distance signaling and resource distribution for growth, development, and environmental responses. The vascular tissues act as long-distance molecular transport conduits, and short-distance molecular exchange is facilitated by intercellular channels called plasmodesmata. Plasmodesmata are cytoplasmic bridges that cross the cell wall to join neighboring cells and allow the movement of soluble molecules. As we have never identified plants without plasmodesmata, we can conclude they underpin a critical element of plant physiology. Furthermore, plasmodesmata are dynamic and appear to act as sluice gates between cells, suggesting that cells differentially benefit from connection and isolation in different scenarios. However, we have little knowledge of the full range of molecules that move through plasmodesmata, and the information and resources that they carry between cells. Thus, the questions of why plasmodesmata open and close, how this controls cell-to-cell traffic, and how it underpins the execution of a response remain unanswered.

With respect to small, soluble molecules, we consider plasmodesmata to be indiscriminate cytoplasmic channels, essentially holes between cells. Thus, any small soluble molecule or ion, unless sequestered in a subcellular compartment or large macromolecular complex, is subject to bulk cytoplasmic flow and diffusion and can move cell-to-cell through plasmodesmata. Smaller molecules move more easily through plasmodesmata than larger ones; small dyes such as carboxyfluoresein diacetate (460 Da) move faster between cells than Green Fluorescent Protein (GFP) (27 kDa), demonstrating a size dependence of plasmodesmal flux. While it is thought there is likely an upper size limit for passage through plasmodesmata (known as the size exclusion limit), variation in mobility of proteins larger than GFP under different conditions suggests it is unlikely that this is a fixed and uniform property across plasmodesmal populations.

There are many plasmodesmata at cell interfaces allowing for a high capacity of plasmodesmata-mediated molecular exchange. However, for some molecules, active transporters also exist through which they can cross the cell membrane and be taken up into neighboring cells from the apoplast. The interaction between this apoplastic transport pathway and the symplastic pathway is increasingly evident as essential to establish observed patterns of distribution for molecules such as the hormone auxin (Mellor et al., 2020; Sager et al., 2020). Indeed, when active transporters exist, it not only allows for integrated transport mechanisms but also presents the possibility that the process of molecular exchange between cells is highly buffered and thus protected, that is, if one pathway is downregulated, the other can compensate as for sugar transport during cotton fiber elongation (Ruan et al., 2004).

**Figure 4:**
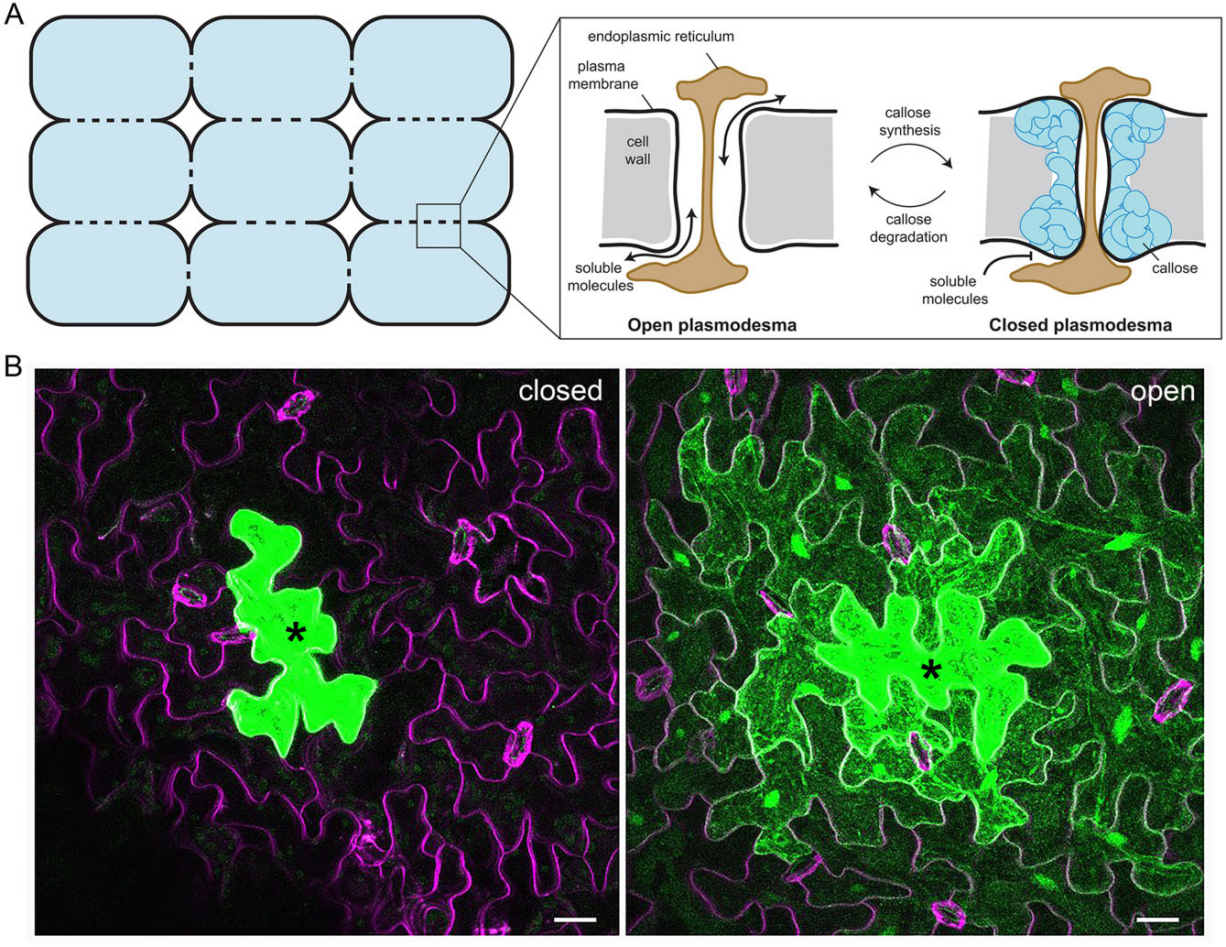
Plasmodesmata are dynamic intercellular connections. A, Plasmodesmata connect neighboring cells and close and open via regulation of callose synthesis and degradation. Adapted from (Maule et al., 2012) under CC BY-NC 3.0 https://creativecommons.org/licenses/by-nc/3.0/#. B, When plasmodesmata close, it limits the intercellular movement of molecules, but we do not understand how this contributes to the success of responses. In these images, GFP (green) is being synthesized in the cells marked with an asterisk. When the plasmodesmata are closed, the GFP is restricted to the site of synthesis (left image) whereas when the plasmodesmata are open, the GFP moves through plasmodesmata into the surrounding cells (right image). Cell outlines are in magenta and the scale bar is 20 μm. Images are maximum projections of confocal z-stacks of Arabidopsis leaf epidermal cells.

Plasmodesmata are dynamic structures, opening and closing under range of conditions via callose deposition and degradation in the surrounding cell wall (Figure 4A). Plasmodesmal closure transiently isolates cells during developmental transitions, such as the initiation of lateral roots (Benitez-Alfonso et al., 2013). This isolation presumably allows changes associated with cell fate determination to occur independently of surrounding cells. An extreme example of cellular isolation in development is the removal of plasmodesmata from the guard cell–pavement cell boundary in the leaf epidermis, presumably to allow the autonomous ion fluxes that define cell shape and control stomatal opening.

Transient plasmodesmal closure and symplastic isolation also occur in response to stresses such as pathogen perception, cold temperatures, wounding, and high concentrations of reactive oxygen species, but the role of symplastic isolation here is not yet well understood. Plasmodesmal closure during immune responses is underpinned by a range of specific proteins that range from plasmodesmata-specific receptors through to callose synthases (Cheval and Faulkner, 2018), and mutants that cannot close their plasmodesmata mount an impaired response (Lee et al., 2011; Faulkner et al., 2013). This identifies plasmodesmal closure as a critical process, but the contribution that plasmodesmal closure makes to an overall immune response is not clear.

Plasmodesmal closure restricts cell-to-cell exchange of soluble molecules, and thus the role it plays in stress responses must relate to a protective benefit from isolation and/or the containment and accumulation of molecules relevant to the response. Stress responses involve the active synthesis of a range of molecules and plasmodesmal closure might serve to concentrate them at the site where they are needed. It is conceivable that this allows signaling molecules such as hormones to accumulate and reach an activity threshold and thus activate localized responses. Upon plasmodesmal re-opening, these molecules might spread to surrounding cells and activate signaling in cells that did not perceive the original stress and thus propagate the response.

Plasmodesmal responses to pathogen signals are early responses, occurring within 30 min of stimulus perception (Xu et al., 2017; Cheval et al., 2020). They rely on specialized machinery and signaling cascades (Stahl et al., 2013; Grison et al., 2019; Cheval et al., 2020), allowing them to be rapidly controlled independently of signaling in the PM triggered by the same stimulus. The speed at which plasmodesmata close suggests that regulation of cell-to-cell connectivity is a primary response, and thus possibly one that later responses depend upon. Further, the specificity of plasmodesmal signaling cascades raises an intriguing question of why plasmodesmal responses are mediated independently? Are plasmodesmata regulated independently because plasmodesmal closure is not always advantageous? The isolation induced by plasmodesmal closure might come at an unaffordable cost for some cells and tissues, e.g., if cells and tissues depend on symplastic supply of nutrients or other resources. In this scenario, independent regulation of plasmodesmal responses provides the possibility that a cell can respond to a stress without inducing the tradeoffs caused by closing plasmodesmata and inducing isolation.

Plasmodesmata are essential to plant growth and physiology, and their dynamics indicate that regulation of the distribution and allocation of soluble molecules is a critical component of cellular responses. While the cytoplasmic path through plasmodesmata is passive and rapid for small, soluble molecules, we have little idea of the full range of mobile molecules that use this pathway to access neighboring and distal cells. Dissecting the identity and function of symplastic traffic remains technically challenging, but knowledge of the molecular information and resources plasmodesmata allow to move both short and long distances, and the processes that are dependent on cell-to-cell exchange, will transform our understanding of how the regulation intercellular connectivity underpins multicellular plant responses.

### Funding

Research in the Faulkner lab is supported by the Biotechnology and Biological Research Council Grant (BB/L000466/1, BBS/E/J/000PR9796) and the European Research Council (725459, “INTERCELLAR”).

## Is there retrograde signaling from vacuoles to the nucleus?

### (Written by Yan Zhang)

Plant vacuoles not only fulfill the roles of lysosomes in digestion and nutrient recycling, but also participate in cell elongation, responses to biotic and abiotic stresses, the storage of proteins or metabolites, and cell death. Vacuoles are dynamic in terms of their number, size, and morphology. Recent progress indicates that vacuolar dynamics, that is, dynamic changes of vacuolar number, size, or morphology, are regulated developmentally as well as in response to environmental cues. This raises the question of whether vacuolar dynamics are solely responses to change, or if they in turn feed back to the nucleus and stimulate change.

Numerous examples of vacuolar dynamics have been documented. In *Arabidopsis thaliana* roots, cells at different stages of differentiation show different vacuolar dynamics: differentiated cells contain fewer but larger vacuoles than those in undifferentiated cells (Cui et al., 2019). Vacuolar dynamics also associate with the first zygotic division, which is essential for the establishment of embryonic pattern in angiosperms. Tubular vacuoles form around the apically migrating nucleus whereas larger vacuoles fill the basal part of an elongating zygote. Such vacuolar dynamics are critical for polar positioning of the zygotic nucleus and for zygote-division asymmetry (Kimata et al., 2019). Dynamic vacuolar rearrangements are landmark events in male and female gametogenesis. Unicellular microspores undergo pollen mitosis I to develop into a bicellular microspore, a process marked by the conversion of a large central vacuole in the unicellular microspores to numerous small vacuoles in the bicellular microspores (Yamamoto et al., 2003). In contrast, the first nuclear division of functional megaspores (FMs) during female gametophytic development is marked by the appearance of a large central vacuole separating a chalazal nucleus (CN) and a micropylar nucleus (MN; Drews and Yadegari, 2002). Vacuolar dynamics have also been extensively studied in guard cells for their role in stomatal movement. Opening of stomata associates with an increased vacuolar volume whereas closure associates with a decreased vacuolar volume, achieved either by vacuolar fission and fusion or by convolution and de-convolution. Other stresses, such as salt, have been reported to induce vacuolar dynamics.

Defects in vacuolar dynamics often result in developmental defects (Gao et al., 2014; Belda-Palazon et al., 2016; Kimata et al., 2019). Mutations of ESCRT, SNARE, homotypic fusion, and vacuolar protein sorting complex, regulators of vacuolar trafficking routes, as well as regulators of vacuolar acidification have been reported to cause embryo lethality, defective male and female gametophytic development, or abnormality in cell division and differentiation of the root meristem (RAM).

**Figure 5:**
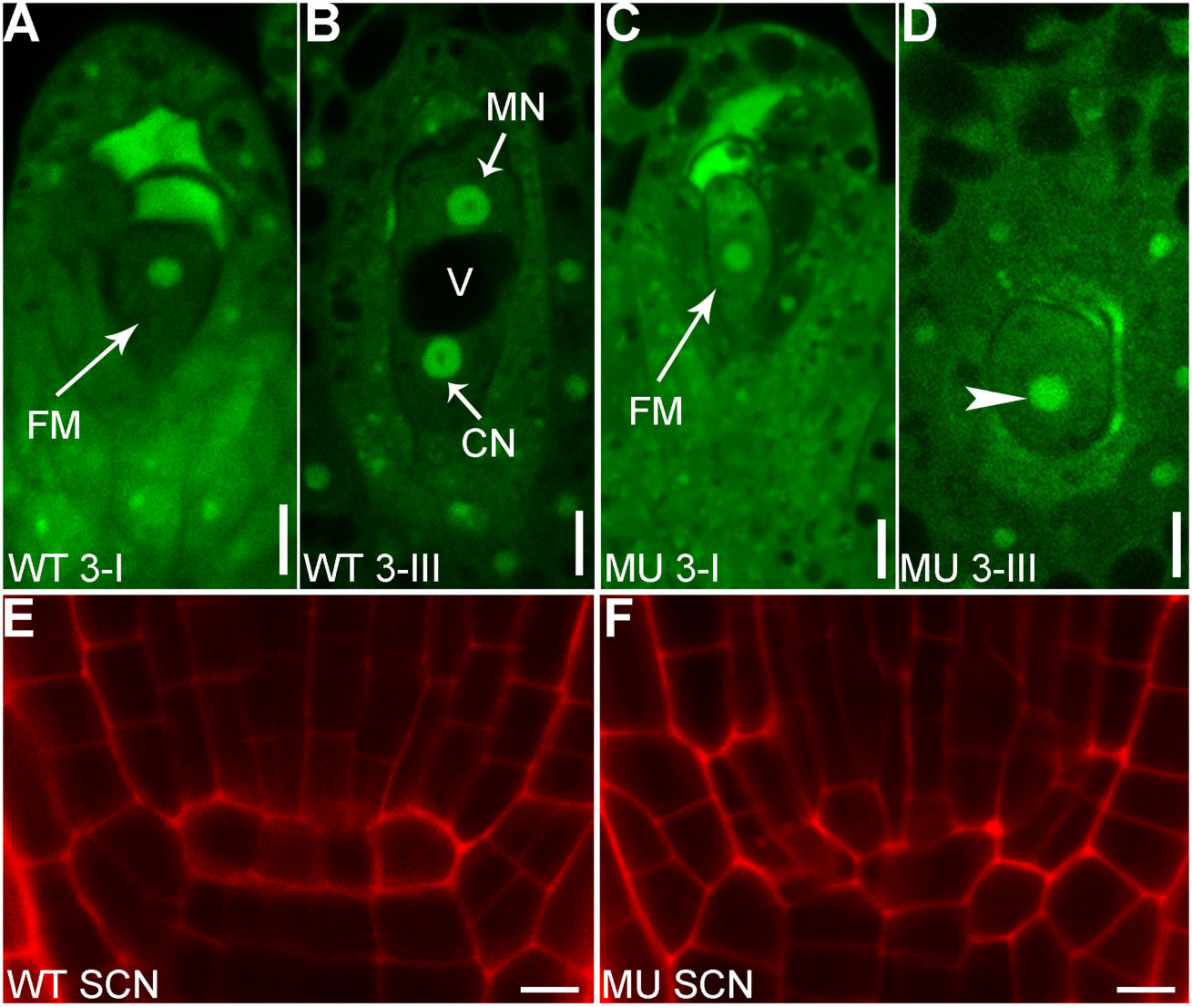
Mutations of a vacuolar regulator cause the arrest of mitotic division during female gametogenesis and the disruption of the stem cell niche (SCN) in the RAM. A–D, Confocal laser scanning microscopy (CLSM) of a wild-type (A and B) or mutant ovule (C and D) at Stage 3-I (A and C) or Stage 3-III (B and D). V, vacuole; CN, chalazal nucleus; FM, functional megaspore; MN, micropylar nucleus. The arrowhead points at the nucleus, which failed to undergo mitosis. E and F, CLSM of wild-type (E) or mutant (F) SCN. Mutations of a vacuolar regulator cause abnormal divisions of the quiescent center. Roots were stained with PI. Bars = 5 μm.

Despite these reports, vacuolar dynamics are usually considered a correlative factor rather than a causative factor in these developmentally regulated processes, which are controlled by nuclear gene expression. However, the possibility of retrograde signaling from vacuolar dynamics to nuclear-controlled cell division and differentiation should be considered. The lethality of male or female gametophytes in mutants of vacuolar regulators often has been shown to be due to mitotic arrest. Defective vacuolar biogenesis in FMs always accompanies the failure of mitotic division (Figure 5) whereas the asymmetric division from unicellular microspores to bicellular microspores fails to occur when vacuolar dynamics are compromised. In other words, defects in vacuolar dynamics cause the mitotic arrest during gametogenesis. Another example occurs in RAM development. Reduced root growth by mutations of vacuolar regulators was often presumed to be caused by reduced cell elongation since vacuoles are critical for turgor-driven cell expansion. However, defective cell division and differentiation of RAM rather than cell elongation were detected in mutants of vacuolar regulators (Figure 5, E and F).

Coordinating different membrane compartments and the activities occurring within them is necessary for the survival of eukaryotic cells and the fitness of the organism. Exciting discoveries in the past decade uncovered retro-signaling pathways from chloroplasts and mitochondria to the nucleus. As the two largest organelles in plant cells, whether vacuoles send retrograde signals to influence nuclear activities is, therefore, an intriguing question to be addressed.

I propose two retrograde signaling routes through which vacuolar dynamics regulates nuclear-controlled processes. Vacuolar dynamics could control membrane targeting of signaling proteins to influence nuclear gene expression. For example, dynamic vacuolar trafficking influences the PM abundance of BRASSINOSTEROID INSENSITIVE 1, PIN-FORMED, and PYR1-LIKE 4 (Gao et al., 2014; Martins et al., 2015; Belda-Palazon et al., 2016), receptors or carriers of brassinosteroid, auxin, and abscisic acid, respectively. Vacuole-mediated changes of their PM abundance or asymmetry are thus able to modulate intracellular signaling of these phytohormones into alterations of nuclear gene expression.

Strictly speaking, vacuolar dynamics that influence PM abundance of proteins to modulate gene expression in the nucleus may not be considered a retrograde signaling route since no molecules are directly transmitted between the two compartments. In this regard, the homeostasis of protons and calcium ions controlled by vacuolar dynamics could provide the second possibility for retrograde signaling from vacuolar dynamics to the nucleus. Vacuoles are the main storage compartments for protons and calcium, two critical ions mediating multiple cellular activities including the activities of transcription factors, either directly or indirectly (Martinière et al., 2013; Schönknecht, 2013; Shen et al., 2013). Vacuolar dynamics would change the abundance or activities of tonoplast ion transporters/pumps to induce fluxes of protons and calcium across the tonoplast. Changes in cytoplasmic concentrations of these ions will in turn influence their levels in the nucleus, which ultimately result in changes of gene expression or of chromatin structures.

Precisely determining whether vacuolar dynamics is a correlative or causative factor in developmentally or environmentally regulated processes has many challenges. For a start, changes of vacuolar number, size, or morphology are usually rapid and occur in their native cellular and developmental context. To visualize vacuolar dynamics in vivo and in real time is therefore challenging. Although genetic mutations have been useful to uncover key roles of vacuolar dynamics in development or environmental responses, it is difficult to distinguish direct consequences of defective vacuolar dynamics from indirect ones. Thus, to develop methods that allow physical or pharmacological manipulation of vacuolar dynamics and to monitor their molecular and developmental consequences is of vital importance. Finally, sensors for protons and calcium that are sensitive to rapid concentration changes within a large concentration range in the vacuolar lumen and in the nucleus have yet to be developed. Despite these daunting tasks, I believe that the first step toward resolving this question is to embrace the possibility of retrograde signaling from vacuolar dynamics to the nucleus and try to answer it little by little.

### Funding

Work on vacuolar dynamics in the Zhang Lab is supported by the National Science Foundation of China (NSFC) (grant number 31625003, 31970332).

## How do root cells accommodate fungal endosymbionts?

### (Written by Maria J. Harrison)

The ability to accommodate intracellular fungal symbionts is surprisingly widespread in the plant kingdom. One of the oldest examples is the endosymbiotic association with arbuscular mycorrhizal fungi, which occurs in >70% of vascular flowering plant species and positively influences plant mineral nutrition as well as carbon distribution below ground (Delaux and Schornack, 2021).

**Figure 6:**
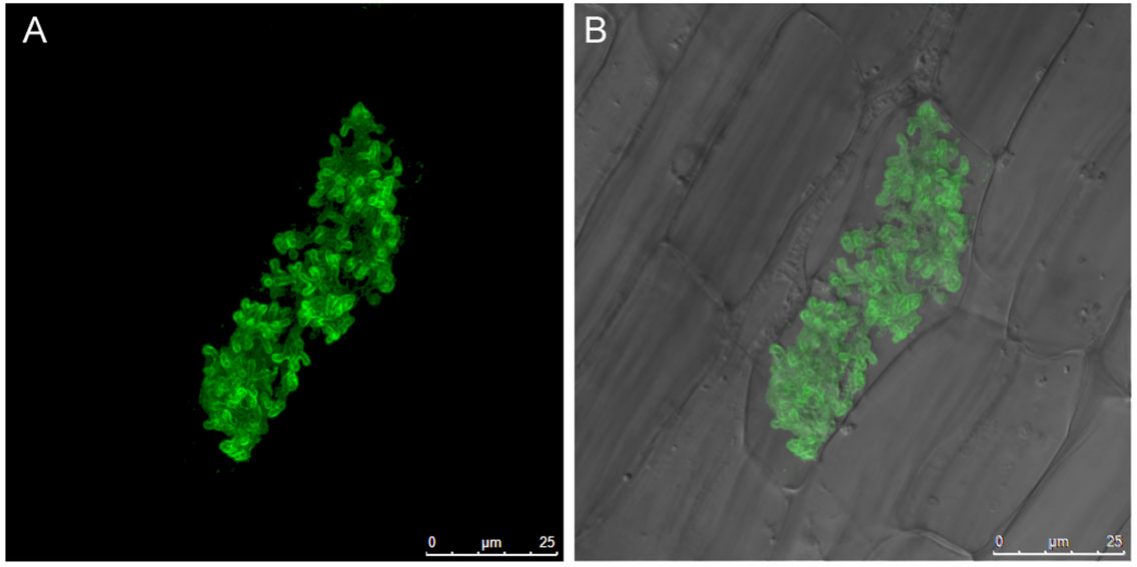
The PAM in a *Medicago truncatula* root cortical cell. Visualized as a consequence of a phosphate transporter-GFP fusion protein located in the membrane. A, GFP image. B, GFP and bright-field images merged. The PAM closely outlines the fungal arbuscule, which occupies a substantial proportion of the cortical cell. The name “arbuscule” is derived from the Latin for small tree.

In an arbuscular mycorrhiza (the name given to the association), the arbuscular mycorrhizal fungus colonizes the root cortex and differentiates within the cortical cells developing highly branched hyphae called arbuscules. Each arbuscule is closely surrounded by a plant membrane, the periarbuscular membrane (PAM), with the result that the arbuscule is housed in an apoplastic compartment within the cell (Figure 6). The narrow apoplast and expansive PAM provide an interface optimized for reciprocal nutrient exchange (Gutjahr and Parniske, 2013; Ivanov et al., 2019; Roth et al., 2019).

From a cell biology perspective, intracellular accommodation of this large endosymbiont is a feat of coordinated cellular rearrangements. These include cytoskeletal alterations, constriction of the central vacuole, endoreduplication, increased cytoplasm, generation, and focal deposition of the PAM whose surface area is two- to five-fold larger than that of the PM, redirection of metabolism to provision the fungus with carbon, and additional proteins for transport across the PAM. The association has fascinated generations of researchers and there is now considerable understanding of the plants symbiosis signaling pathway, downstream transcriptional regulators, metabolic changes, and nutrient transport (MacLean et al., 2017; Choi et al., 2018). However, there are still many mysteries; in particular, we lack an understanding of how the profound cellular alterations are achieved.

Within the root cortex, the first cellular changes are initiated as the hypha approaches a cortical cell. Calcium oscillations, predictive of an active symbiosis signaling pathway are visible in the nucleus (Sieberer et al., 2012) and a cytoplasmic aggregation of ER, Golgi bodies, and cytoskeletal elements, termed a prepenetration apparatus (PPA), accumulates in the root cell adjacent to the hyphal contact point (Genre et al., 2005). The PPA appears critical for hyphal growth into the cell and it does not assemble in symbiosis signaling pathway mutants, but how symbiosis signaling results in the PPA is unknown. Endoreduplication also occurs in these cortical cells and may be an output of symbiosis signaling (Carotenuto et al., 2019), but again, how this leads to endoreduplication is unknown.

With the PPA in position, the fungal hypha then penetrates the cortical cell wall; how it manages to do this is also an open question as arbuscular mycorrhizal fungi lack cell wall degrading enzymes (Tisserant et al., 2013). Perhaps, the PPA facilitates this through focal secretion of cell wall loosening enzymes or local adjustments to wall pH. Following traversal of the wall and a short period of intracellular linear growth, the hypha starts to branch extensively, often by repeated dichotomous branching at the hyphal tips (Bonfante-Fasolo, 1984; Figure 6). What triggers this dramatic shift in growth form is entirely unknown but it occurs only in the cortical cells.

Development of the PAM begins with a small invagination of the PM around the tip of the hypha and is likely facilitated by the secretory elements of the PPA, but we lack a molecular understanding of this phase. As the hypha starts branching, the tip number rapidly increases and the PAM grows by exocytosis ahead of each new branch tip. This is a cellular juggling feat, requiring focal secretion at an exponentially increasing number of locations. Several proteins required for this phase of PAM development have been identified, including an EXOCYST subunit, EXO70I (Zhang et al., 2015), VAPYRIN, a protein of unknown function (Feddermann et al., 2010; Pumplin et al., 2010; Murray et al., 2011) and membrane fusion proteins, VAMP721d/e (Ivanov et al., 2012), and SNAREs (Huisman et al., 2016; Pan et al., 2016). However, the nature of the signaling that directs focal membrane deposition around the growing hyphal tips is unknown. EXO70I locates adjacent to the membrane at each hyphal tip and could provide a spatial landmark for assembly of the EXOCYST, but if so, how is EXO70I recruited to these locations? A role for plant GTPases might be anticipated but so far none have emerged as significant in this context.

Actin filaments and microtubules reorient and accumulate densely in the vicinity of the developing PAM (Genre and Bonfante, 1998; Blancaflor et al., 2001). Actin is likely involved in vesicle trafficking to the PAM but so far, the molecular details are missing. Complexes such as SCAR/Wave that regulate actin polymerization (Yanagisawa et al., 2013), have not yet been linked to PAM development.

A particular unusual feature of the PAM is that it grows in an inward direction into the root cell (as an analogy, think of inward tip growth with a continually increasing number of tips) (Harrison, 2012). It seems plausible that the inwardly-directed growth is driven by pressure from the growing hyphal branches, and potentially enabled by a reduction in turgor pressure within the root cell as the central vacuole constricts. How modifications to vacuole size and morphology are coordinated with the arbuscule and PAM growth remains to be determined. Maybe there is a role for auxin, as colonized root cells have increased auxin levels (Lauressergues et al., 2012) and auxin can regulate vacuole morphology (Scheuring et al., 2016). The hyphal branches of the arbuscule must also alter the mechanical stresses on the cell which could in turn influence cortical microtubules and provide polarity cues (Hamant et al., 2019), so perhaps mechanical signaling has a role to play in the development of the PAM.

Development of an intracellular compartment to accommodate arbuscular mycorrhizal fungi is a remarkable process, even more so when we consider that the arbuscules are short-lived (<5 days) and the majority of the cellular alterations are subsequently reversed. The cytoplasm is withdrawn from the arbuscule branches resulting in their collapse (Bonfante-Fasolo, 1984), and the remnants, along with the apoplastic matrix and ultimately the periarbuscule membrane is degraded, potentially by the action of host cell hydrolases (Floss et al., 2017). The vacuole re-establishes its central location and the microtubules reassemble a typical orderly oblique array. There is still much to learn about the processes by which root cells host their intracellular fungal symbionts and in doing so, we not only gain mechanistic insights into endosymbiosis, but also into the cellular plasticity of root cells.

### Funding

Research in the author’s lab is supported by the National Science Foundation (NSF IOS-2139351) and the Office of Science (BER), US Department of Energy (DOE-SC0014395).

## What is the role of cell edges in plant morphogenesis?

### (Written by Charlotte Kirchhelle)

**Figure 7:**
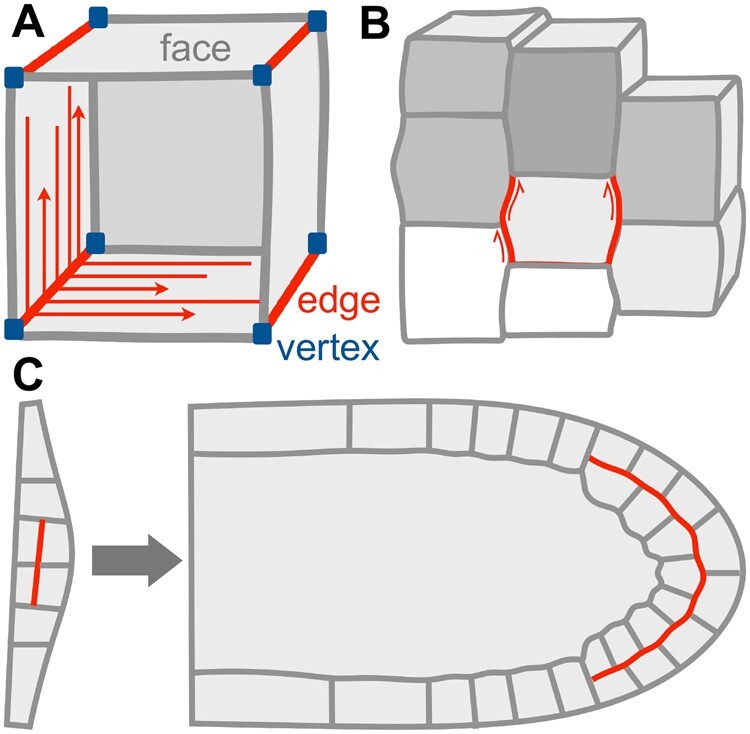
Cell edges in morphogenesis. A, At the subcellular scale, edges can provide directional information to establish anisotropy at cell faces, for example, through nucleation or local stabilization of microtubules (red arrows). B, Cell edges can accumulate stresses (red arrows) arising at the cellular and supracellular scale through differential growth in adjacent cells. C, Cell edges can act as persistent polarity landmarks during organogenesis: For example, periclinal cell edges established in early lateral root development (red line, left) retain their relative position in the tissue during subsequent organ development (red line, right).

The striking polyhedral shape of plant cells inspired Robert Hooke to coin the term “cell” in 1665, and generations of scientists after him to produce mathematical descriptions of plant cell geometry and growth (Thompson, 1917; Korn, 1982). While some plant cells can attain complex geometries with undulating walls as they differentiate, plant cells start their life as polyhedra made up of faces, edges, and vertices (Figure 7A). How plants form their diverse and intricately shaped organs from these simple shapes is a central question in biology. Notably, an early model of plant growth explicitly considering geometric features predicted that the rate of growth at cell edges was primary and causal for growth control (Korn, 1982). However, it did not provide an explanation of how the requisite growth determinant at edges may function or be positioned. This phenomenological approach was also at odds with more popular strategies aiming to gain a mechanistic understanding of morphogenesis, which gave rise to the current view that morphogenesis depends on the integration of genetic, biochemical, and mechanical factors across multiple spatio-temporal scales, from the sub- to the supracellular.

Intriguingly, cell edges have subsequently emerged from experimental studies of morphogenesis as biochemically and mechanically distinct domains (Jarvis et al., 2003; Ambrose et al., 2011; Kirchhelle et al., 2016; Yoshida et al., 2019; Elliott and Kirchhelle, 2020), reinvigorating the concept of cell edges as domains of growth control. Our understanding of the role of cell edges during morphogenesis is still in its infancy, but below I summarize recent developments and open challenges related to two pertinent questions:

### Why are cell edges important?

Conceptually, cell edges are notable features from a topological, geometric, and mechanical perspective.

In plants, cells are fixed in their position by their surrounding cell wall, and tissue topology only changes through cell division. Once formed, cell edges are therefore persistent features and, in tissues undergoing stereotypic division patterns, can even retain positional specificity throughout organogenesis (Figure 7C). Recently, members of the SOSEKI protein family have been found to accumulate and persist in the periphery of specific cell edges at different developmental stages (Yoshida et al., 2019), which suggests that cell edges may act as long-term polarity landmarks.

Geometrically, cell edges delimit cell faces, for which they can act as directional information sources (Figure 7A). Because stiff cellulose microfibrils constrain growth parallel to their net orientation, the core paradigm of plant growth control predicts that organized cellulose deposition along anisotropic microtubule arrays promotes directional growth (Green, 1980). Cell edges can influence the organization of microtubule arrays at cell faces: edges pose a physical barrier to microtubules for geometric reasons, and have also emerged as sites of protein-mediated microtubule nucleation (Ambrose and Wasteneys, 2011), stabilization (Ambrose et al., 2011), or de-stabilization (Takatani et al., 2020). Consequently, selective positioning of microtubule-regulating proteins at specific edges can contribute to the formation of directional microtubule arrays.

Cell edges are conspicuous from a mechanical perspective: first, the high turgor pressure in plant cells creates significant mechanical stresses specifically at cell edges (Jarvis et al., 2003). Second, cell edges also connect cell faces on the same or adjacent cells that can differ significantly in their growth rate and pattern, which may lead to a build-up of shear stresses at the edge domain (Figure 7B). Stress accumulation may explain a need to reinforce cell edges mechanically, and there is ample evidence plants can indeed specifically modify their cell wall at edges. Cell edges in mature tissues are often enriched in de-methylesterified pectins and/or phenolic compounds, which can stiffen the cell wall through cross-linking (reviewed by Elliott and Kirchhelle, 2020). The establishment of biochemically distinct cell walls at edges implies localized deposition of cell wall materials and their associated biosynthetic machinery. Although cell wall composition in growing cells is less well characterized, these cells specify a transport pathway to their cell edges through the small GTPase RAB-A5c (Kirchhelle et al., 2016). This edge-directed transport route has been implicated in directional growth control, possibly through local modification of cell wall mechanics (Kirchhelle et al., 2019).

### How are cell edges specified?

Edge-polarization can be cytoskeleton-dependent: edge-directed transport depends on intact actin and microtubule cytoskeletons, and RAB-A5c patterning changes when microtubule organization is altered (Kirchhelle et al., 2019). On the other hand, polar SOSEKI localization is not sensitive to depolarization of the cytoskeleton (Yoshida et al., 2019) but instead depends on SOSEKI polymerization to form localized protein patches (van Dop et al., 2020).

However, neither of these mechanisms explains how plant cells identify their edges per se or differentiate between edges within the same cell. Below I offer two speculative mechanisms that may account for this:

#### Membrane curvature

Membrane curvature can be sensed through a variety of (often simultaneously active) mechanisms, including preferential recruitment of lipids to regions of different curvatures and recruitment of curvature-sensing proteins (Jarsch et al., 2016). A curvature-sensing mechanism could explain both the identification of edges in principle, and the differentiation of old from newly formed edges, which differ in their curvature.

#### Mechanical stress

Another possibility relates to the previously mentioned accumulation of stresses at edges: these may not only be a constraint that cells need to ameliorate, but could simultaneously be involved in specifying the cell edge domain through the recruitment or activation of mechanosensitive proteins. Stresses are expected to differ at different edges depending on cell size, shape, and differences in growth rate between neighboring cells, and could thus also account for differential specification of different edges. A stress-based mechanism would be particularly advantageous for factors involved in modifying the cell wall at edges to maintain cell wall integrity.

In summary, molecular advances in recent decades have demonstrated that plant cells identify their cell edges as distinct spatial domains during morphogenesis, confirming the phenomenological prediction that cell edges are important features of growth control. This brief overview offers some suggestions for why cell edges may be important and how they may be specified. Exploring these questions further will be essential for answering the ultimate open edge question: how edge-based growth control is integrated into the complex multi-scale system controlling morphogenesis.

### Acknowledgments

I am grateful to Dr Liam Elliott for critical reading of the manuscript.

### Funding

I acknowledge funding from the Biotechnology and Biological Sciences Research Council (BB/P01979X/1) and the Leverhulme Trust (ECF-2017-483).

## How is the cell division site determined?

### (Written by Gohta Goshima)

**Figure 8:**
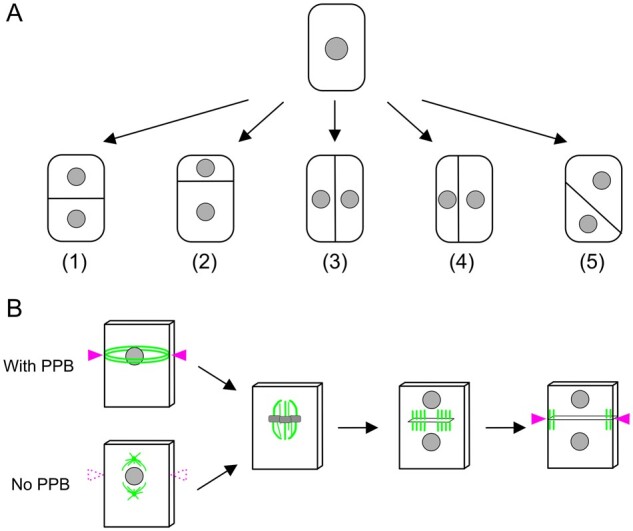
A cell decides where it divides. A, Cell division could occur symmetrically (1, 3) or asymmetrically (2, 4, 5). The orientation of the division plane could also vary. B, PPB marks the future division site prior to spindle assembly (arrowheads). Phragmoplasts and the associated cell plate expand toward the marked site. However, the division site can be defined without PPB at least in some cell types (bottom). How this is achieved is largely unknown. One possibility is that the division site is marked without the aid of microtubular PPB (dotted arrowheads). The formation of MTOC in the absence of PPB has been observed in moss gametophores. Microtubules are colored green; chromosomes are in gray.

When students who just learned about cell division are asked to draw a simple diagram of the final step of cell division, many would instantly place the division site in the middle of the cell and perpendicular to the cell’s long axis (Figure 8A (1)). However, in nature, this is not always the case. Division sites can be off-centered and/or oriented in different ways (Figure 8A (2–5)). The positioning of the division site is a critical issue for living organisms. For example, an off-centered division site is a hallmark of asymmetric division, which underlies the development of multicellular organisms.

Regardless of the final location and orientation, the positioning of the division site is usually an active rather than a passive, random process. In plants, the key determinant of the process is the preprophase band (PPB), which is formed prior to mitosis (Rasmussen et al., 2011). At G2 phase of the cell cycle, cortical microtubule arrays are reorganized into a bundle called the PPB (Figure 8B). A bipolar spindle is then assembled such that the spindle axis is perpendicular to the plane of the PPB, via the action of microtubules bridging the PPB and the spindle. The PPB is disassembled during prometaphase, with several proteins left behind, which define the division site at the cortex. Upon sister chromatid separation, a microtubule-based bipolar structure called the phragmoplast is assembled. The phragmoplast expands and is guided precisely to the cortical division site (i.e. former position of the PPB), while recruiting cell plate materials.

This prevailing mechanism for division site determination is intriguing from two viewpoints. First, this is completely different from what is known in several well-studied animal model cells, which form spindles of similar size and shape to plants (e.g. *Caenorhabditis elegans* embryos, *Drosophila melanogaster* neuroblasts, and human tissue culture cell lines). In these systems, microtubules generated radially from the centrosome (missing in plants) interact with the cell cortex and exert pushing or pulling force (Kiyomitsu, 2015). The final spindle position is determined via the balancing of forces involving astral microtubules from two centrosomes. The oocyte, which is a rare acentrosomal animal cell, also utilizes a different mechanism from plants, as it has no equivalent to plants’ cortical microtubule arrays, PPBs, and guidance mechanism (Almonacid et al., 2014). The lack of similarity is noteworthy, considering the overall conservation of mitotic genes and mechanistic analogy of the cell division process between animals and plants (Yamada and Goshima, 2017). How the plant-specific mechanism has evolved is an outstanding question (Buschmann and Zachgo, 2016).

Second, even within the plant kingdom, there are multiple cell types in which cortical microtubules or PPBs are missing, such as endosperm cells of angiosperms or protonemal cells of the moss *Physcomitrium patens* (Olsen, 2001; Livanos and Müller, 2019). This has been known for a long time, but not much attention has been paid: most studies on division site determination have focused on PPB. However, it was recently reported that an Arabidopsis mutant that does not form any discernable PPB only suffers from some loss of precision in division plane orientation and essentially undergoes normal development (Schaefer et al., 2017). PPB-independent mechanisms must be present in many plant species. What are they? In moss, the importance of cytoskeleton-based mechanisms in processes such as nuclear positioning, acentrosomal formation of the microtubule-organizing center (MTOC), spindle orientation, and spindle motility, has been recently elucidated (Umeda et al., 2021). The question is whether or not these mechanisms function cooperatively with the PPB-based mechanism. If yes, how? Another possibility is that cells that do not possess a PPB might have analogous, but microtubule-independent, cortical markings (Figure 8B, bottom). The marks would communicate in some way with the mitotic apparatus to dictate cell division placement. In this scenario, microtubules simply help to make the PPB more robust, which might explain the minor cell division defects in the Arabidopsis mutant lacking the PPB.

Another interesting aspect of division site determination is its plasticity according to the influence of the neighboring cells. Hormones, mechanical stress, and cell shape change have been known to influence division plane selection (Livanos and Müller, 2019). This is not a plant-specific issue; how extrinsic forces affect centrosome position, spindle orientation, and division site is an active research area in animals (van Leen et al., 2020). A possible scenario in plants is that mechanical stress affects the localization of cell polarity factors as well as cortical microtubule array orientation, which leads to PPB positioning (Zhang and Dong, 2018). How this mechanism, which is dissimilar to that found in animal cells, has evolved and is integrated with the PPB-independent mechanism is an open question.

Questing for a mechanism underlying an essential and complex cellular process is great fun for cell biologists. While the mechanisms of cell division may have been largely elucidated and turn out to be remarkably conserved in eukaryotic lineages (McIntosh and Hays, 2016), division site determination certainly remains on the fun list. It is reasoned that lineages with cell walls could have fundamentally different mechanisms from those that have no walls. Even within the plant kingdom, the mechanism might be diverse. Only by collecting the data from a wide variety of cell types could we get a full picture on division site determination mechanisms.

## What are the emergent effects of polyploidy on the biology of the cell, and how are any such “rules” conditioned by cell type?

### (Written by Jeremy E. Coate and Jeff J. Doyle)

**Figure 9:**
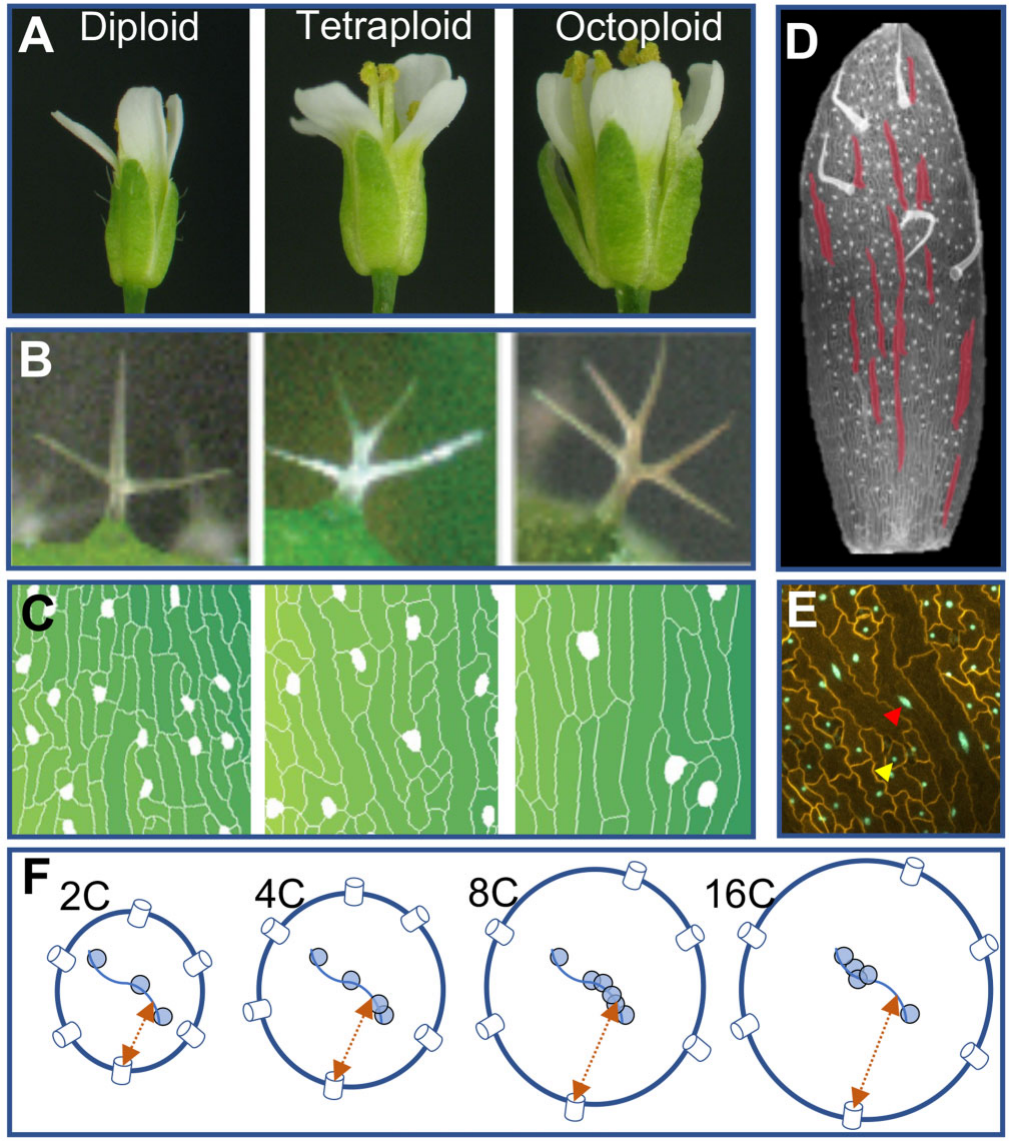
Ploidy induces nucleotypic changes in size. A, Arabidopsis flowers increase in size with polyploidy. B, Arabidopsis sepal trichomes increase in size and degree of branching with polyploidy. C, Sepal abaxial epidermal cell area increases, and density of guard cells (white) decreases, with ploidy. D, Within a single organ (sepal epidermis), cell size varies dramatically due to differences in ploidy resulting from programmed endoreduplication (16C sepal giant cells shown in red, diploid guard cells appear as white ovals). E, Nuclear volume increases with increasing cell size induced by endoreduplication. Yellow and red arrows indicate the nuclei of a diploid and an endopolyploid epidermal cell, respectively, in the sepal epidermis (cell walls stained with propidium iodide shown in yellow, nuclei expressing ML1:H2B-GFP, an epidermis-specific nuclear marker, shown in green). F, Simplified diagrams of nuclei from diploid, tetraploid, octoploid, and endopolyploid 16C cells (left to right), illustrating nucleotypic changes in nuclear size, density of nuclear pores (blue cylinders on nuclear surface), chromatin compaction (a strand of DNA in the nuclear interior shown as a line with nucleosomes shown as blue circles), and distances from sites of transcription to nuclear pores (orange arrows). The effects of polyploidy on chromatin accessibility can vary from locus to locus. A–C, Images within panels are shown at the same scale. A–E, Adapted from Robinson et al. (2018), Figure 3. Copyright American Society of Plant Biologists.

Size is a critical parameter in cell biology. Distance, concentration, surface/volume ratio, chromatin compaction, and other mechanical stresses—all are affected by size of cells and organelles. It is widely accepted that cell types—however, one defines this elusive term (Clevers et al., 2017)—have a “right size” (Ginzberg et al., 2015), which is somehow sensed and which constrains cell growth, division, differentiation, and function. Cell and nuclear size are correlated strongly with genome size (Figure 9). These and other “nucleotypic” phenotypes (phenotypes influenced by the amount of DNA per cell rather than by any specific DNA sequence) such as duration of the cell cycle may derive from biophysical laws (Bennett, 1971), but cause and effect for these seemingly emergent properties of genome size have yet to be teased apart (Doyle and Coate, 2019).

Polyploidy, by doubling or multiplying the genome, is a major source of genome size increase, and is a ubiquitous process in biology and evolution. Many cell types undergo developmentally programmed endoreduplication (genome replication in the absence of mitosis)—an estimated 90% of herbaceous angiosperms have endopolyploid cells in most tissues (Scholes and Paige, 2015)—and endopolyploidy is also characteristic of cancers and other disorders (Shu et al., 2018). For programmed endopolyploid cells, increased size may be associated with their function.

When failure of mitosis leads to stem cells or gametes with doubled chromosome complements, polyploid organisms can result (Mason and Pires, 2015). Such whole organism polyploidy is particularly prevalent in plants, and has occurred throughout their history. Genome mapping and sequencing studies have revealed that all flowering plant lineages are fundamentally polyploid: a recent phylogenomic study identified nearly 250 independent polyploidy events in approximately 1,000 species surveyed, with the genomes of most major lineages showing evidence of around four whole-genome duplications or triplications since the plant common ancestor (One Thousand Plant Transcriptomes Initiative, 2019). “Diploid” *A.* *thaliana*, for example, with its small genome and low chromosome number, is historically 96-ploid. It has been estimated that 15% of all flowering plant speciation events involve polyploidy (Wood et al., 2009).

It has long been hypothesized that cell size increase is the key event leading to the many documented changes in physiology, anatomy, morphology, and development of polyploids (Muntzing, 1936), that culminate in such ecological phenomena as increased invasiveness (te Beest et al., 2012). But what underlies cell size increase remains poorly understood. Although some cell types are well known to increase in size with increased genome size (e.g. guard cells, Beaulieu et al., 2008), this is not universally true and may vary across species. For example, Katagiri et al., 2016 found that, unlike epidermal pavement cells, Arabidopsis palisade cells showed only a weak response to polyploidization, yet across a range of flowering plant species palisade cells increased in size with increased genome size (Théroux-Rancourt et al., 2021). The relationship between cell size and polyploidy—endopolyploidy, whole organism polyploidy, and the interaction between the two phenomena—thus remains mysterious, even contentious, and can vary even among genotypes of a species (Tsukaya, 2019; Pacey et al., 2020). Additionally, the functional consequences of this relationship at the tissue and organ level are further complicated by the phenomenon of compensation, whereby increases in cell size are often at least partially offset by decreases in cell number (Hisanaga et al., 2015; Robinson et al., 2018; Doyle and Coate, 2019). Thus, several fundamental questions remain unanswered. What are the mechanisms connecting cell size to genome size? What controls the observed variation (among cell types, tissues, and species) in this response? What are the functional consequences of size increases and their variation, within the cell and at higher levels of organization? Tackling these questions has the potential to inform broader questions about cell type-specific factors affecting cell size, as well as constraints imposed at the tissue or higher levels.

Autopolyploids can be synthesized de novo in the laboratory by doubling a diploid plant, and these autopolyploids will differ from the diploid at the molecular level only in DNA content and gene dosage (Figure 9). Autopolyploidy is an epigenomic macromutation (Doyle and Coate, 2020), but its effects, from gene expression to phenotypes, are relatively subtle (Corneillie et al., 2019; Doyle and Coate, 2019). Autopolyploids have the same cell types as do their diploid progenitors, but increased size of cells and/or nuclei presumably affect a host of cellular processes such as chromatin compaction, transit time of messenger ribonucleoproteins from transcription to the nuclear pore complex, and macromolecular crowding (Figure 9F). What are the phenotypic manifestations, if any, of such size effects? Because any such perturbations occur in an otherwise isogenic context, synthetic autopolyploids should be excellent models for studying cellular homeostasis in response to size increases.

Increased size of the cell and its component parts is, arguably, the closest thing to a “rule” that polyploidy follows, though this response varies by cell type, tissue and species. Elucidating the causes and consequences of these size increases is fundamental to understanding how polyploidy has engendered novelty at higher levels of organization, and will, more broadly, shed new light on the biology of cell types, the regulation of cellular processes, and mechanisms of cellular homeostasis.

## Can mechanical forces trigger new cell fates in plants?

### (Written by Olivier Hamant)

Building on a corpus of knowledge from the 19th century as well as more recent developments in molecular genetics, micromechanics, live imaging, and computational biology, there is today no doubt that mechanical forces, and/or their consequences, play an instructive role in morphogenesis. This is notably the case for cell wall synthesis, cell division plane orientation, vasculature patterning, organ motion, and growth (Echevin et al., 2019). However, these conclusions rather relate to a role of mechanical signals on structural aspects of morphogenesis, that is, only maintaining existing identities. Can mechanical signals also trigger new identities?

So far, mechanical feedback rather appears to act downstream of biochemical signaling (e.g. auxin). There is evidence that mechanical stress can also act, at least in part, upstream of hormone patterning. This is notably the case for the auxin efflux carrier PIN-FORMED1 for which recruitment at the PM depends on membrane tension (Nakayama et al., 2012), and for which polarity may reflect mechanical conflicts between adjacent cells (Heisler et al., 2010). However, this role seems rather minor, when considering that local ablations or mutants with mis-shaped meristems exhibit few phyllotactic defects. It is as if mechanical cues would add robustness to patterning and identity, rather than triggering new identities. Maybe the root, and its flexible lateral organogenesis could provide more conclusive evidence of an organogenetic role of mechanical cues (Ditengou et al., 2008; Lucas et al., 2013; Abu-Abied et al., 2015).

Similarly, gene expression has been found to depend in part on mechanical signals. This includes the *TOUCH* genes, which role in cell fate remains to be established since the corresponding mutants have very mild phenotypic defects, and no clear defect in identity (Lee et al., 2005). The activity of the *SHOOT MERISTEMLESS* promoter was found to depend on mechanical signals at the shoot apical meristem, independent of PIN1-dependent auxin patterning. Yet, mechanical perturbations could only increase the expression of *STM* in sites where it would be normally expressed, that is, mechanical perturbations did not alter a robust prepattern (Landrein et al., 2015).

Disorganization of cortical microtubules and associated mechanical isotropy of the walls could lead to ectopic gene expression at the shoot apical meristem, opening the prospect that cells could modify their identity in response to mechanical cues (Armezzani et al., 2018). Recently, a more direct impact of a cell wall defect on the patterning of root hair and flowers was revealed using the cellulose synthase inhibitor isoxaben. The associated pathway involves the wall integrity sensor STRUBBELIG (Chaudhary et al., 2021). Whether the initial trigger is biochemical, mechanical or a combination of both remains to be investigated.

**Figure 10:**
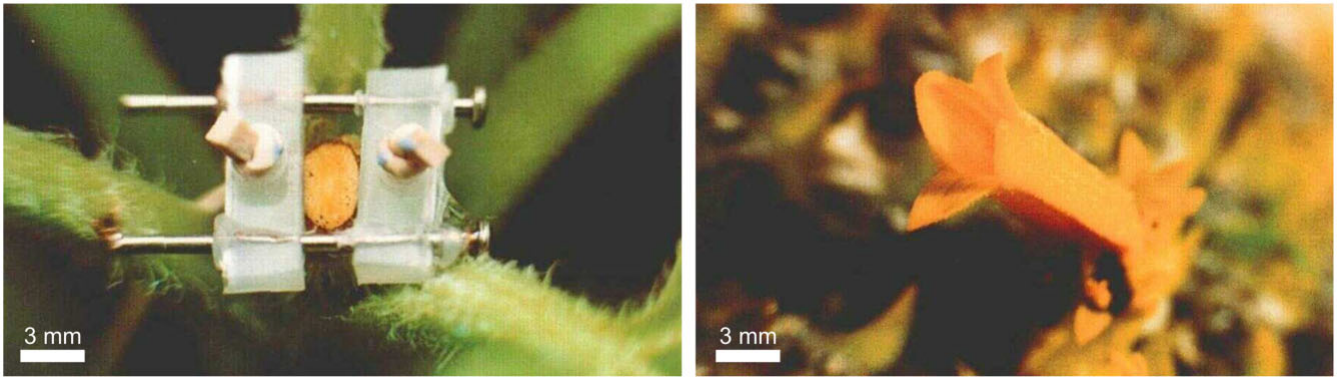
Altered patterning and fates upon compression in sunflower capitulum. Left: A young capitulum is constrained for several days with a vice. Scale bar: 5 mm. Right: Observation of floret with the symmetry of a central floret, but with the dimension of a ray floret, from a constrained capitulum. Scale bar: 3 mm. Adapted from Figure 7, A and D in Hernandez and Green (1993). Copyright American Society of Plant Biologists.

The search for a role of mechanical stress in triggering new fates may go back to Paul Green’s earlier work on sunflower. In particular, when compressing a sunflower capitulum in a vice, the identity of the florets was affected. The number of bracts was altered, and some ray florets became radial (Figure 10;  Hernandez and Green, 1993). However, this pioneering study remains phenomenological, and would certainly deserve to be revisited with current molecular tools.

To find an established role of mechanical forces in triggering ectopic gene expression and cell fates, one actually needs to read the literature in the animal kingdom. Maybe the best established, yet still debated, case of mechano-induction of gene expression is that of *TWIST*, a gene required for mesoderm differentiation in Drosophila. Not only could the expression of this gene be induced by a suite of mechanical perturbations and by the naturally occurring compression during gastrulation (Farge, 2003; Desprat et al., 2008), but its homolog, *NOTAIL*, could also be induced in zebrafish during epiboly, a type of embryonic cell movement (Brunet et al., 2013). Even more striking was the observation that in both phyla, the induction of the gene involved the same mechanotransduction pathway (β-catenin; Farge, 2003; Brunet et al., 2013). As a take-home message, deciphering the role of mechanical signals in triggering new identities will require the identification of the relevant mechanotransduction pathways. As plant scientists, we may get some inspiration from other kingdoms. For instance, calpain is a cytosplasmic protease acting downstream of PIEZO, a calcium mechanosensitive channel and central mechanosensory in animal development. In Arabidopsis, calpain is encoded by a single gene called *DEFECTIVE KERNEL1* (*DEK1*) and it contains a large transmembrane domain, which is associated with a calcium mechanosensitive channel activity (Tran et al., 2017). Knowing that plant epidermises are often under tension, and that *dek1* mutants do not specify their epidermis correctly, the putative role of DEK1 in force-dependent cell fate specification may be a promising research avenue (Malivert et al., 2018).

The identification of genetic targets of mechanical signals may in fact lead us to revisit the mechanistic roots of gene expression. Gene expression is very much a structural problem. For instance, among the factors involved, one finds topoisomerases, gyrases, and helicases, that is, factors that function to modify the physics of chromatin (Redding, 2021). Similarly, if the chromatin status depends on compaction, then a force may be sufficient to affect that status (Stephens et al., 2019). Some actually propose that forces are the first-order control of chromatin remodeling, before biochemical modifications at discrete sites (Pagliara et al., 2014). In plants, the chromatin state can be correlated to the mechanical status of the cell. In particular, hyper-osmotic conditions make chromatin more compact (Goswami et al., 2020) and mechanical compression at the organ-meristem boundary in the shoot apical meristem also correlates with modifications in chromatin marks and compaction (Fal et al., 2021). The question of the role of mechanical signals in triggering new identities may thus call for the broader question of synergies between biochemical and mechanical cues in cell fate determination: to what extent is gene expression and cell fate coupled to the mechanical status of the tissue, the cell, and the nucleus?

## How does a single differentiated somatic cell reprogram and gain pluripotency?

### (Written by Keiko Sugimoto)

When we, or other mammals, get injured, the best our bodies can do is to heal the remaining damaged tissue as we lack the ability to regenerate new arms or legs. Plants, in contrast, display amazingly diverse forms of regeneration. Most notably, beyond merely repairing tissues, plants can produce new shoots or roots from wound sites when injured in certain ways. Given that most somatic cells have taken on a specific developmental fate, they need to undergo some degree of reprograming, that is, a change of cell fate, and reacquire the potential to develop new organs. Over the last few decades, we have made substantial progress in understanding how plant somatic cells reprogram and gain pluripotency (Ikeuchi et al., 2019). It is now well established that stress caused by wounding generates signals important for initiating cellular reprograming, and furthermore, how plant cells translate such signals into reprograming cues is becoming increasingly clear (Hoermayer and Friml, 2019; Ikeuchi et al., 2020). Additionally, we know that plant hormones strongly enhance regeneration in vitro, and the molecular mechanisms governing auxin- and cytokinin-directed cellular reprograming are becoming defined (Mathew and Prasad, 2021).

**Figure 11:**
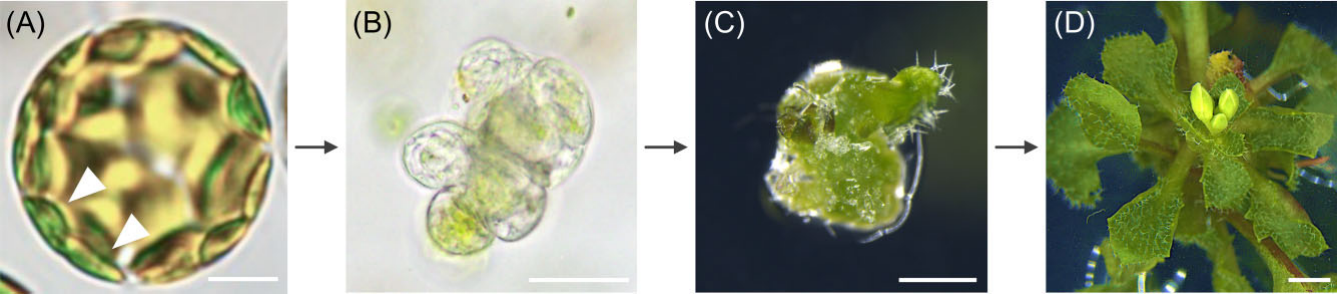
Regeneration of a whole plant from a single leaf mesophyll protoplast. A, A protoplast isolated from a leaf mesophyll cell. B, Protoplast cells that have undergone several rounds of cell division. C, Shoot meristem formation from protoplast-derived callus. D, A regenerated plant. Arrowheads in (A) mark a chloroplast. Scale bar = 10 mm (A), 50 mm (B), 1 mm (C), 2 mm (D) (Photos courtesy of Yuki Sakamoto).

While recent work has revealed that plant regeneration does not always involve reprogramming of differentiated cells, dedifferentiation is clearly important for regeneration in at least some contexts (Ikeuchi et al., 2016). In Arabidopsis, for instance, in vitro callus formation starts from division of pericycle cells, which despite being found in somatic tissue still retain high organogenic potential (Atta et al., 2009; Sugimoto et al., 2010). It is also true, however, that differentiated somatic cells contribute to regeneration in other contexts and one striking example that highlights the astonishing developmental flexibility of plant cells is the regeneration of whole plants from individual leaf mesophyll protoplasts (Figure 11;  Takebe et al., 1971). Differentiated plant cells have a fully expanded vacuole, with the nucleus squeezed into a thin layer of cytoplasm found at the periphery of the cell. Since reprograming of differentiated cells is usually accompanied by reactivation of cell division, there must be a mechanism by which the vacuole deforms and allows the nucleus to relocate to the correct division plane within a cell. Further complicating matters, some differentiated cells have gone through endoreduplication, an alternative cell cycle during which cells replicate chromosomes without undergoing cytokinesis, and thus have polyploid nuclei. We know that endoreduplicated cells can reinitiate cell division (Ikeuchi et al., 2015), but how they manage to separate the polytene chromosomes is completely unknown. In addition, differentiated cells often have highly specialized organelles, such as chloroplasts found in mature leaf cells, which they must be able to lose in order to acquire a new fate. How can cells erase these features associated with differentiation and subsequently redifferentiate, thus taking on a new cellular state?

As discussed above, it is likely that a combination of stress-activated signaling and hormone signaling promotes reprograming of differentiated cells. Given that forced expression of reprograming regulators or key developmental regulators is sufficient to induce division of differentiated cells (Ikeuchi et al., 2015), these early signaling events may help activate factors that drive developmental transitions and reinitiate cell division. This situation will be analogous to the induced pluripotent stem cells in mammals since ectopic expression of a set of genes can induce pluripotency in differentiated cells (Takahashi and Yamanaka, 2006). What we do not know, however, is how these developmental regulators, which are typically transcription factors, are integrated into a gene regulatory network that can coordinate subcellular remodeling in such a way that brings mitotically inactive cells back into the cell cycle. Is activation of standard cell cycle machinery sufficient to reinitiate the cell cycle? Or do differentiated cells require additional mechanisms to deal with subcellular properties unique to them? Cell cycle reactivation likely involves reorganization of the cytoskeleton, but how do cells interpret information transduced through the gene regulatory network and orchestrate cytoskeletal changes? We also do not know how organelles are lost during dedifferentiation. Is this actively regulated? If so, how is regulation of this linked with cell cycle reinitiation?

Addressing these questions has been a challenge since these reprograming events happen infrequently, and thus they have not been amenable to characterization using cell or molecular biology techniques. With recent advances in automated live imaging technology as well as various single-cell resolution analyses, however, it might now be possible to start tackling some of these questions. Untangling the manner by which differentiated plant cells reprogram may also help decipher what is unique in plant cells compared to mammalian cells in this respect and, importantly, what mechanisms prevent reprograming of differentiated mammalian cells.

### Acknowledgments

I am grateful to Yuki Sakamoto, Hatsune Morinaka, and David Favero for their comments on this manuscript.

### Funding

The work in my laboratory is supported by grants from Ministry of Education, Culture, Sports, and Technology of Japan (20H03284 and 20H05911).

## How does polarity develop de novo in isolated plant cells?

### (Written by Liam Dolan)

Robert Bloch defined polarity as a “change or gradation in character [that] occurs along the axis from one end to the other” (Bloch, 1943; Sinnott, 1960). He distinguished two types of polar systems. The polar axis may exist within an isolated, single cell where the character-state at one end of the cell differs from the other end, which he termed unicellular polarity. Alternatively, the axis may occur across a group of cells, organ, or organisms where the character state on one side of the cell group differs from the character state at the other, which he termed multicellular polarity. Bloch hypothesized that auxin may be involved in the development of multicellular polarity. Discoveries over the past 30 years have defined the molecular mechanisms of auxin-mediated multicellular polarity in developing groups of cells such as embryos, shoots, roots, and leaves. However, little more is known about the development of polarity from the unpolarized state in isolated plant cells—unicellular polarity—than was reviewed by Bloch in 1943 (Bloch, 1943; Sinnott, 1960).

**Figure 12:**
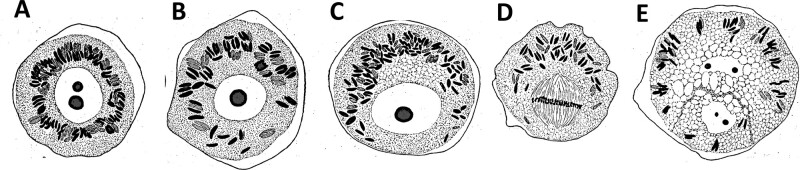
De novo polarization of Equisetum spores. A, Unpolarized germinating spore with no obvious polar axis. The nucleus is the white disc at the cell center with two dark nucleoli. The light source was at the top of the figure. B, Organelles (mainly chloroplasts) are cleared from the shaded (lower) side of the cell. C, The nucleus is located in the shaded (lower side) of the cell from which other organelles are largely excluded. D, Mitotic spindle located in the shaded (lower) side of the cell. E, After cytokinesis a larger cell is located on the illuminated (upper) side and a smaller cell located on the shaded (lower) side of the two-celled sporeling. The light source was located at the top of the figure. Modified from Nienburg (1924) Die Wirkung des Lichtes auf die Keimung der Equisetum spore. *Ber Deutsch Bot Ges* 42, 95–99 with permission. © 1924 Deutsche Botanische Gesellschaft/German Botanical Society.

Experiments on isolated cells demonstrated that environmental factors direct the development of unicellular polarity. One of the best examples reviewed by Bloch (1943) is a series of experiments on the development of polarity in the spores of horsetails (*Equisetum* species). Spores are the haploid cells produced by meiosis that are surrounded by a mechanically resilient sporopollenin-rich wall called a sporoderm. Bloch reviewed evidence that indicated that these cells do not have an inherent polarity (Figure 12). Instead, polarity develops at germination and is controlled by the direction and intensity of incident light (Stahl, 1885; Nienburg, 1924). Spores incubated on damp media swell, forming a sphere without any obvious polarity. If spores are exposed to unidirectional light, the cytoplasm becomes polarized without a change to the spherical shape of the cell. Larger organelles (including chloroplasts) are cleared from the shaded side and concentrate on the illuminated side of the cell (Nienburg, 1924). A mitotic spindle forms near the clear zone with one pole located on the illuminated side and the other on the shaded side. The new cell wall that forms during cytokinesis is located toward the shaded side of the cell. It has an hour-glass shape and at its center, it is oriented perpendicular to the direction of the incident light. The cell that develops on the shaded side is smaller than the cell that develops on the illuminated side because the new cell wall forms near the clear zone. The smaller cell differentiates as a rhizoid and undergoes no further cell division while the apical cell is a regenerative stem cell that divides to form the entire body of the plant. This demonstrates (1) how a polar axis develops de novo within a single cell and then (2) how unicellular polarity is transformed into a multicellular polar axis through cell division and the inheritance of polar cues. The unicellular polarity of the germinating spore is inherited and directs the formation of the multicellular axis in the horsetail gametophyte. Given the role of auxin in the formation of polarity in multicellular groups, it is likely that auxin is involved in the elaboration of the axis in the multicellular state. However, it is unknown how unicellular polarity is generated in the first instance.

There is evidence that the development of cell polarity from an apolar state is different from the mechanisms by which polarity is inherited in a daughter cell produced by mitosis. Mechanisms for the inheritance and maintenance of preexisting unicellular polarity after mitosis have been discovered in yeasts where the polarity of mother cells determines the polarity of daughter cells. For example, the two ends of a *Schizosaccharomyces pombe* (fission yeast) rod-shaped cell are different; one is older than the other, having been formed one cell cycle apart. Consequently, as soon as a fission yeast cell forms it is already polarized and this polarity is propagated from one cell generation to the next. The mechanism by which polarity is inherited from one cell generation to the next is understood in great detail.

There are few reports that define the mechanism for the establishment of the polarized cell state from an unpolarized state (Makushok et al., 2016). Upon starvation, fission yeast develops apolar resting cells (Makushok et al., 2016). On return to nutrient media (starvation exit), these cells polarize and start to grow. The mechanism of de novo polarity formation has been discovered by observing these resting cells as they develop polarity on starvation exit. The first step is the formation of spatially random patches of sterol-rich domains throughout the PM. The Tea1p, a polarity-determining factor, then accumulates in these sterol-rich membrane domains and is required for their polar localization to two sites on the cell surface. Factors required for both polarity maintenance and growth then accumulate at these two sites where polar cell extension subsequently occurs to form the rod-shaped cell. This polarized state is maintained, but not initiated, by a mechanism requiring Cdc42p, a conserved Rho family GTPase required for the maintenance of cell polarity across the eukaryotes (Adams et al., 1990; Etienne-Manneville and Hall, 2002). The establishment of the polarized state from an apolar state involves an initial random distribution of multiple sterol-rich membrane domains in an apolar cell which is reduced to two sites where growth-promoting factors and polar elongation occurs. This polarity is then maintained by a mechanism that requires Cdc42p and inherited in subsequent cell generations.

What is the mechanism that controls the transition of an unpolarized single plant cell to the polarized state in the absence of inherited cues? It is likely that incident light is the cue that directs polarity (Stahl, 1885; Nienburg, 1924). There is evidence from moss spores and regenerating moss protoplasts that red and blue lights are required for the establishment of polarity (Mohr, 1956; Jaffe and Etzold, 1965; Cove et al., 1978, 1996). It is unknown how light signaling establishes the molecular asymmetry that underpins the development of polarity. There is evidence from diverse species that the ROP family is required for the late stage of polarity maintenance as shown for Cdc42p in fission yeast (see e.g. Cheng et al., 2020; Makushok et al., 2016). However, it is unknown if ROP proteins or their regulators are involved in the de novo establishment of polarity in isolated plant cells. Furthermore, there is evidence that BREAKING OF ASYMMETRY IN THE STOMATAL LINEAGE (BASL) protein, which is localized in a polar manner in many cell types, accumulates on one side of isolated, regenerating Arabidopsis protoplasts which initially lack polarity (Dong et al., 2009; Chan et al., 2020). This suggests that regenerating protoplasts develop polarity de novo and BASL is a marker of this polarity, but the role of BASL in this process remains to be defined. Identification of mechanisms that control the development of spore polarity in organisms like horsetails, where unicellular polarity develops from the unpolarized state may provide an answer.

### Funding

Research in my laboratory is funded by the European Research Council Advanced Grant (Contract 787613) and the Austrian Academy of Sciences.

## What is the spectrum of cellular functions for membraneless organelles and intrinsically disordered proteins?

### (Written by Heather Meyer and David W. Ehrhardt)

**Figure 13:**
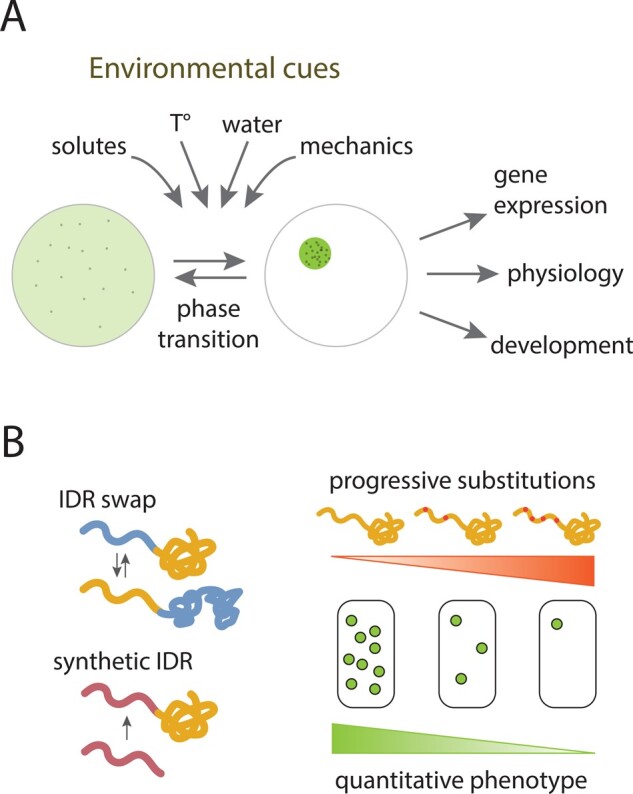
Examining the drivers and functions of IDP phase separation. A, IDPs undergo reversible liquid–liquid phase separation to form MLOs under different environmental cues, which likely function to facilitate a range of cellular, developmental, and physiological processes under changing environmental conditions. B, Two methods that can be used to assess the specific function of IDP phase separation: (left) swapping the IDR from a native IDP with either a heterologous or synthetic IDR that is known to phase separate and (right) quantitatively tuning IDP phase separation behavior and testing if it quantitatively tunes the respective phenotypic output.

Researchers have long understood a protein’s specific function through its tertiary structure and dynamics. Yet, proteins with low sequence complexity that lack a defined tertiary structure—intrinsically disordered proteins (IDPs)—are known to make up ∼30–50% of eukaryotic proteins, causing a re-examination of the structure–function paradigm (Peng et al., 2014). IDPs have become of increasing interest in recent years due to their remarkable ability to undergo liquid–liquid phase separation, a property that arises out of their structural fluidity at physiological temperatures, and ability to self-associate through weak multi-valent interactions (Posey et al., 2018). Phase separation of IDPs has been shown to underlie the formation of membraneless organelles (MLOs)—including well-known cellular compartments such as the nucleolus, Cajal bodies, P-bodies, and the algal pyrenoid—which likely serve to partition and regulate discrete biochemical reactions including transcription, translation, signaling cascades, and carbon fixation (Cuevas-Velazquez and Dinneny, 2018). Interestingly, the self-assembly of IDPs is sensitive to environmental conditions such as solvent concentration and composition, pH, and temperature (Posey et al., 2018; Figure 13A). As all organisms require the ability to perceive and respond to their environment; the question arises as to whether evolution has exploited the environmental sensitivity of IDPs phase separation for driving new functions—such as sensors or direct conduits—in order to regulate critical cellular, physiological, and developmental responses. A further question is if IDP functionality primarily involves assembly into phase-separated liquids and gels or if there are other important functions for these disordered peptide sequences.

Plants are an excellent system for investigating the functional spectrum of IDP phase separation because their sessile nature requires them to integrate and remember perceived environmental conditions to adjust their physiology and make life history decisions. Additionally, plant cells form environmentally sensitive MLOs that harbor IDPs or proteins with extensive intrinsically disordered regions (IDRs), which are accessible for observation in the intact organism (Emenecker et al., 2020; Meyer, 2020). Nevertheless, testing the functionality of phase separation at the molecular level remains challenging for two primary reasons: (1) IDPs’ lack of amino acid conservation across phylogenetic space and (2) difficulties in untangling structural effects from other functions those residues may participate in. Yet, new molecular tools and techniques are providing opportunities to interrogate the role of environmentally driven phase separation in plant cellular and physiological responses. For example, the replacement of native IDRs with physiochemically similar synthetic and/or heterologous IDRs may enable the functional testing of phase separation versus specific amino acid sequences (Hastings and Boeynaems, 2021; Figure 13B). Additionally, new CRISPR technology, such as CRISPR-GO—a technique able to reposition genomic loci in relation to nuclear bodies (Wang et al., 2018)—may be used to test the dependence of MLO function at precise locations by repositioning phase-separated compartments away from their putative site of action in living cells. Even techniques like proximity protein labeling (Mair et al., 2019) can be used to investigate the challenging and often transient fluid composition of MLOs in order to gain new insights into possible MLO functions.

IDP phase separation may alternatively be assessed by quantitatively correlating changes in phase manipulation with plants’ environmentally driven phenotypes (Figure 13B). Experimental and computational studies have revealed that changing the intra and intermolecular bonds, concentration, or solvent solution of IDPs may affect phase separation behavior in a modular and programmable way (Garcia Quiroz et al., 2019; Dzuricky et al., 2020; Moses et al., 2020; Yu et al., 2021). These studies unlock opportunities to manipulate phase behavior along different experimental axes while measuring phenotype, providing more robust tests for associating phase separation with function. Additionally, they provide new approaches for designing plants with re-programmable growth and development regimes under different abiotic and biotic conditions.

The discovery that IDPs and proteins with IDRs phase separate within living cells have prompted the emergence of a new field, leading to unprecedented insight into the role of IDP phase separation behavior and the formation of MLOs. However, we must also consider that there may be a wider range of possible IDP functionalities other than phase separation. For instance, multivalent interactions may not only drive phase separation but instead may act as flexible linkers for hub protein–protein interactions (Thieulin-Pardo et al., 2015). Additionally, the conformational fluidity of IDRs may serve as dynamic tentacles for the recruitment and concentration of other molecules to the site where they are tethered (Thieulin-Pardo et al., 2015). Therefore, a careful assessment of IDP behavior on sub-cellular, cellular, and organismal levels will be required to determine the functional spectrum of phase separation and other putative roles facilitated by IDPs.

### Acknowledgments

We would like to acknowledge all of the research that we were unable to cite due to the short format nature of this perspective.

### Funding

This work was supported by the Simons Foundation-Life Science Research Foundation [Meyer LSRF18/Carnegie Fund 10861] and the Carnegie Institution for Science.

## How do plants deal with internal noise?

### (Written by Arezki Boudaoud)

**Figure 14:**
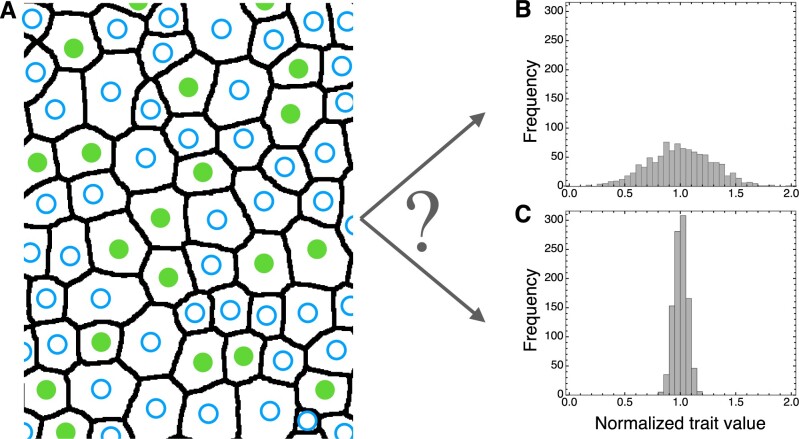
How do plants deal with internal noise? A, Tissue with cells that stochastically express a gene of interest; transcription is on in green-filled nuclei and off in empty blue nuclei. B and C, Histograms of two traits with low (B) or high (C) standard deviation (hypothetical histograms for traits quantified over 1,000 individual plants). Does cell-to-cell variability (A) lead to variable phenotypes (B) or to precise phenotypes (C)? Examples of variable traits include germination time, number of petals in Cardamine, and number of secondary stems in Arabidopsis. Examples of robust traits include sepal size and number of petals in Arabidopsis. Traits such as plant height or rosette diameter in Arabidopsis may be variable or robust, depending on growth conditions.

Natural selection operates on phenotypes, while heritability of traits involves genotypes. Therefore, efficacy of selection requires a well-defined genotype-fitness map. It is tempting to conclude that developmental stability (also termed developmental robustness), that is, the insensitivity of traits to microenvironment (growth conditions), is selected for because the same phenotype is consistently achieved for a given genotype (Hall et al., 2007), leading to robust (i.e. precisely determined) traits (Figure 14). In contrast, as I will detail below, molecular processes are intrinsically random, as illustrated by variability in gene expression (Figure 14). As a consequence, plants face internal noise, that is, all the noisy internal processes emerging from random molecular events, which raises several questions. How do plants deal with internal noise? When are traits robust? Can internal noise be beneficial to the plant?

Cellular processes that involve relatively small numbers of elements appear stochastic, that is, random. Consider for instance exocytosis. At a given location at the plasma membrane (PM), vesicles are delivered intermittently. Indeed, click-chemistry indicates a punctuate pattern of pectin delivery to the cell wall in roots of *A.* *thaliana* (Anderson et al., 2012), implying that exocytic vesicles have reached sparse spots at the PM. However, over long-time intervals, all the PM is reached by vesicles. Over several hours, patterns of pectin delivery appear homogenous (Anderson et al., 2012). A seemingly stochastic process is averaged out in space and time: spatiotemporal averaging yields a uniform pattern, when both the region of interest is much bigger than the spots and observation time is much longer than the typical delay between delivery of two vesicles at a given location of the PM.

Gene expression is also variable in space and time, which may be ascribed to the on/off nature of transcription (Figure 14). Stochasticity in gene expression was first described in bacteria and was ascribed to a combination of external and internal noise (Elowitz et al., 2002). Progress in the live imaging of plants has revealed seemingly random gene expression in a multicellular context. Ubiquitous promoters, whose expression was thought to be uniform, show five-fold spatial and temporal variations (Araújo et al., 2017). Similar variations were observed for the levels of transcription factor *A.* *thaliana* MERISTEM LAYER1 (ATML1; Meyer et al., 2017). If spatial averaging of gene expression occurred, gene expression would appear much less variable at tissue or organism scales than at cell scale, implying for instance consistent gene expression across individual plants. Cortijo et al. (2019) analyzed genome-wide variability in gene expression between individual Arabidopsis seedlings grown in the same conditions (Cortijo et al., 2019). They identified hundreds of highly variable genes, with a standard deviation of expression that is comparable to mean of expression, indicating the lack of spatial averaging for these genes.

Finally, cell growth is variable in space and time. For instance, five- to ten-fold variations in relative areal expansion of cells occur in the shoot apical meristem of Arabidopsis (Uyttewaal et al., 2012) and in Arabidopsis sepals (Hong et al., 2016). Based on computational modeling of sepal growth, on analysis of spatiotemporal variability in cell growth, and on quantification of mature sepal shape, Hong et al. (2016) found that spatiotemporal averaging of growth occurs: sepals are made of thousands of cells that grow with relatively independent fluctuations (variations around average growth), enabling spatial averaging. Cell growth fluctuates over a few hours, while the sepal grows to a mature size in more than a week, enabling temporal averaging to occur. The outcome is sepals that have robust (precise) shape and size (Hong et al., 2016).

Is cell-to-cell variability merely a by-product of noisy, stochastic molecular process or does it have a function? Variability in ATML1 accumulation is a trigger for differentiation into giant cells in Arabidopsis sepals (Meyer et al., 2017). Variability in cell growth was proposed to enable gradients in growth rate across the boundary between flower and shoot apical meristem in Arabidopsis and facilitate boundary formation (Uyttewaal et al., 2012).

Are there specific mechanisms that buffer (i.e. decrease the effects of) cell-to-cell variability to ensure developmental robustness? Mechanisms of interest could act on the parameters of spatiotemporal averaging. For instance, Hong et al. (2016) screened for mutations that affect developmental robustness of flowers. They found that mutating the *FtsH4* gene encoding for a mitochondrial protease increases the level of reactive oxygen species in sepals and decreases cell-to-cell variability in growth and cell wall mechanics with respect to wild-type. This reduces spatial averaging and yields less robust sepal size and shape. Sangster et al. (2008) generated lines in which *HEAT-SHOCK PROTEIN 90* (*HSP90*) is downregulated, which led to an increase in variability of the length of stem between the five first siliques compared to wild-type, suggesting that HSP90 buffers variability in stem growth (Sangster et al., 2008).

In other cases, developmental stability is reduced by internal noise. A salient feature of seeds is variability in germination time, which enables a fraction of seeds to remain dormant and survive through unfavorable environmental conditions. Johnston and Bassel (2018) used stochastic differential equations to model the gene network that regulates production and degradation of abscisic acid (Johnston and Bassel, 2018). They found that this network does not buffer molecular noise and maintains variability in abscisic acid level, which is associated with variability in germination time. In *Cardamine hirsuta*, petal number is variable, unlike the invariant four-petalled flowers in the closely related Arabidopsis. Monniaux et al. (2018) found that the difference in stability of petal number is due to the evolutionary divergence of the transcription factor APETALATA1, which may leave internal molecular noise unbuffered in Cardamine (Monniaux et al., 2018).

How can we make sense of these differences in the buffering of internal noise? A possible answer relies on natural selection. Optimizing fitness may increase or decrease developmental stability, depending on the trait considered. Hall et al. (2007) analyzed developmental stability across Arabidopsis accessions using the number of flowers as a proxy of fitness (Hall et al., 2007). Their results indicate that stability in number of secondary stems is selected for, whereas stability of plant height and rosette diameter is counter-selected in short-day growth conditions. This decrease in the stability of stem length might be explained based on a computational model of phyllotaxis that accounts for stochastic noise in flower initiation (Mirabet et al., 2012). Increasing flower number (and fitness) can be achieved by reducing the time interval between initiation of flowers, which, due to internal noise, increases the number of flowers initiated simultaneously. As a result, variability in internode length and, consequently, variability in plant height are increased.

As can be seen from the above, there are many gaps in the study of internal noise and of its links with developmental robustness. What do we need to make further progress? It would be most informative to simultaneously monitor expression of several genes of interest, cell growth, cell trafficking, and, for instance, cell metabolic state. Mutant screens for loss of developmental robustness or use of natural variability in levels of robustness will allow us to dissect the links between internal noise and developmental stability. Plant fitness also depends on biotic and abiotic stresses, which raises questions about developmental stability in the context of how plants cope with stress. Finally, we note that models enable the investigation of hypotheses linking observations at different scales or about different traits, easing the understanding of the multi-factorial, and sometimes counterintuitive, features of developmental robustness.

## How does order emerge in cells and propagate to organs and organisms from complex dynamical processes?

### (Written by Carlos Messina and Adrienne H. K. Roeder)

The beauty of a snowflake and the coordinated flight of a flock of birds are complex patterns that emerge from the actions of individual water molecules and birds. The interactions of these individuals without any kind of blueprint seem to spontaneously generate ordered systems with properties not present in any of the individual parts—a phenomenon known as emergence. Emergence is a fundamental property of complex systems and ubiquitous in biology (Trewavas, 2006). For example, the interactions of tubulin dimers give rise to the dynamic instability of microtubules and the interactions of these unstable microtubules give rise to complex arrays optimized for the growth of the plant cell. Likewise, the stochastic bursts of transcription in individual nuclei give rise to a predictable increase in total *HEAT SHOCK PROTEIN 101* mRNA in the tissue upon heat shock (Alamos et al., 2021). Considering the myriad of chemical reactions occurring at the subcellular level, in all the cells in organs and organisms, it is fascinating how order emerges from these many chaotic reactions. Understanding and predicting emergent behavior is one of the greatest challenges facing biologists today.

The phenotypic expression of plants and their ability to adapt to changing environments results from the interaction between biomolecules, organelles, organs, and the environment in multidirectional ways. The cell phenotype is determined by the behavior of both macromolecules and organs; in other words, both upward and downward causation are determinants of emergence (Mazzocchi, 2008). Biological “components” often fail (e.g. cells die), are not well understood, and operate nonlinearly, yet reproducible plant phenotypes emerge, such as properly shaped leaves. General principles of modern engineering were developed for systems where components are virtually fault-free, their behavior is understood and held in isolation, and operate in linear response to inputs. Application of these “fault free” principles is limiting plant scientists’ ability to successfully engineer complex plant systems. To engineer important traits with the aim to increase nutritional security and environmental quality, we must learn to engineer emergence.

**Figure 15:**
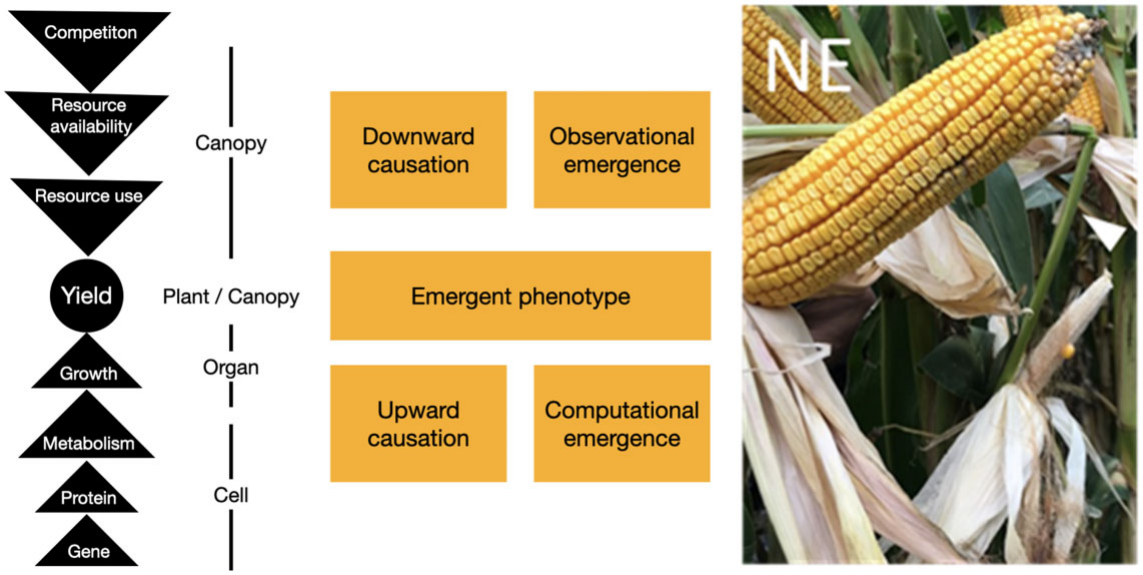
Barrenness is an emergent phenotype resulting from downward causality and competition. Maize phenotypes observed in agriculture production fields managed to attain record yields in Nebraska. White arrow indicates dominated and barren plant.

Emergence can be classified as computational or observational (Figure 15). Computational emergence describes the system when we can deduce the behavior of the whole from the collective dynamics of the lower-level constituents (Baas and Emmeche, 1997). Computational emergence is the implicit paradigm guiding systems biology and molecular and cell biology (Albert, 2007). However, some emergent properties fundamentally cannot be deduced or predicted from their lower-level constituents, which is called observational emergence. The dependence of a protein concentration at time *t* on the concentration of the same and/or other proteins at time *t*−1, can lead to stable states, unstable states, cycles, or chaos (a behavior so unpredictable it appears random because it is highly sensitive to initial conditions).

Lorenz (1995) discovered chaos while studying the atmosphere (Lorenz, 1995). The butterfly effect, where minor disturbances in the atmosphere in one geography may lead to major atmospheric events elsewhere, is a metaphor for the sensitivity of complex systems to initial conditions. Chaos makes observational emergence fundamentally impossible to predict from the behavior of the parts. Only through the lens of chaotic behavior can we understand why kernels seem to randomly abort and produce barren ears in the most productive crop systems in the world (Figure 15). Observational emergence is the implicit concept in crop systems biology (Hammer et al., 2019) where functional relations and phenomenological modeling across levels of organization (e.g. Chew et al., 2014; Messina et al., 2018) fill the void left unexplained by computational emergence. Here we will discuss two emergent phenotypes. Circadian oscillations are an example of computational emergence, the behavior of which we can model at the molecular level predictively. We also discuss crop yield, as an example of observational emergence, in which outcomes cannot be explained solely from a bottom-up perspective.

Circadian oscillations in many biological processes are an example of computational emergence. Organ expansion and growth, water influx, and expression of genes coding for proteins involved in cell wall expansion, cellulose synthesis, auxin transport, and water movement through pores display coordinated diurnal patterns (Harmer et al., 2000; McClung, 2006). An internal clock underpins the orchestration within and across levels of organization. The clock is a system of genes and proteins, and connections among these that create a series of interlocking feedback loops. Protein–protein interactions enable the cell to sense the environment to train the clock and to transduce signals to orchestrate gene expression and cell metabolism (McClung, 2006; Farré and Weise, 2012). Multiple control mechanisms enable the system to oscillate steadily and predictably. mRNA and metabolites were implicated in the regulation of core components of the clock itself to create a robust control system (Staiger and Green, 2011; Farré and Weise, 2012). Recent studies have also shown that there is self-organization for cross-clock synchronization within and across cells (Riedel-Kruse et al., 2007; Masuda et al., 2017). By achieving coherence across cells, the organism prevents chaos and maintains homeostasis (Koronowski and Sassone-Corsi, 2021). Stability in the core oscillator and signaling mechanisms enables the organism to generate regular patterns and adaptations to changing environmental conditions, exemplifying computational emergence.

In contrast to the stability of the clock, changes in photosynthesis at the subcellular level do not translate well to yield across environments due to observational emergence. South et al. (2019) demonstrated ∼40% biomass increase by engineering the glycolate metabolism pathway to alleviate photorespiration in tobacco (*Nicotiana tabacum*) plants (South et al., 2019). However, this biomass increase does not necessarily translate into grain yield across environments. Hammer et al. (2019) used a cross-scale crop model (metabolism-to-crop; Wu et al., 2019) to evaluate how the reported changes in photosynthesis may translate to wheat productivity across environments (from dry to well-watered; Hammer et al., 2019). The largest simulated yield difference for well-watered high yielding environments is ∼10% gain. In many water deficit environments yields differences were zero or even negative. In the case of this simulation study, increased photosynthesis led to increased canopy growth and self-shading, so that the control of photosynthesis at the canopy level was more regulated by interception of light rather than biochemical reactions (Wu et al., 2019). In water-deficit environments, increased photosynthesis and growth lead to increased soil water extraction early in the growing season and consequently intensified water deficit during reproductive stages of development when small differences in water availability at critical times such as flowering can be exponentially amplified to generate ∼50% difference in yield (Messina et al., 2019; Cooper et al., 2020). These are examples of downward causation that can explain the missing biomass and illustrates observational emergence.

We strive to answer questions on order, chaos, and emergence to engineer desired emergent phenotypes. The examples above suggest a gradient of capabilities to engineer systems depending on the balance between computational and observational emergence on the determination of the phenotypes of interest. The difficulty of applying this framework to design plants is illustrated by Simmons et al. (2021) who reported that ∼1% of >1,600 genes evaluated in field trials tested positive at (*P* < 0.1) for improved yield in a large two-decade industry research program (Simmons et al., 2021). Emergence engineering (Krakauer, 2019), whose principles of design assume partial information, computational, and observational emergence, nonlinear responses to inputs, and adaptability in the system, can introduce a paradigm shift in systems biology.

Hammer et al. (2019) offer a thesis upon which to develop an emergence engineering framework for the plant sciences that includes modeling of processes from biochemical to crop level of organization, accounting for upward and downward causality and emergence, and enabling designs that target the aggregate behavior of components (e.g. flowers and leaves), the distributions of outcomes (e.g. yields across environments), and reward functions to harness adaptation (e.g. evolution of breeding populations; Hammer et al., 2019). By applying concepts of emergent engineering encapsulated in crop models and genomic prediction it was possible to consistently improve drought tolerance in maize (Cooper et al., 2020). Emergent engineering is an evolving framework to both advance scientific understanding of biological processes and design biological systems to address societal needs.

### Funding

Research in the Roeder lab is supported by the National Institute of General Medical Sciences of the National Institutes of Health under Award Number R01GM134037 to AHKR and the National Science Foundation (NSF) IOS 1553030 to AHKR.

*Conflict of interest statement*. The authors have no conflicts of interest to declare.

## Notes

The author responsible for distribution of materials integral to the findings presented in this article in accordance with the policy described in the Instructions for Authors (https://academic.oup.com/plcell) is: Adrienne H. K. Roeder (ahr75@cornell.edu).
